# Metallurgical Properties of Biocarbon in Ferroalloy Production—A Review

**DOI:** 10.1021/acsomega.4c00866

**Published:** 2024-05-28

**Authors:** Yu Han, Merete Tangstad

**Affiliations:** † Department of Materials Science and Engineering, Norwegian University of Science and Technology, 7034 Trondheim, Norway

## Abstract

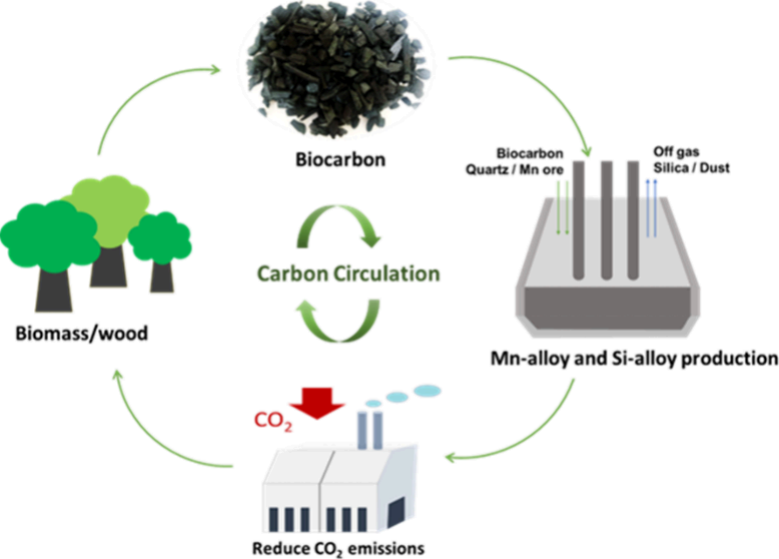

The significant volume of CO_2_ emissions contributes to global warming, which has drawn substantial attention. Metallurgical processes contribute to around 30% of these emissions, with ferroalloy smelting alone equivalent to the collective mean CO_2_ emissions from 11.8 million people. Biocarbon emerges as a promising substitute for fossil reductants, and its research and industrial application have the potential to significantly curtail emissions on a relatively short time scale. As a result, extensive research has been conducted on biobased carbon materials and their practical utilization in metal production processes. In this review, an overview of the methodologies employed to assess the CO_2_ reactivity, electrical conductivity, reactivity toward slag and SiO, and mechanical strength is illustrated. The impact of characterizations on its behavior within furnaces is concluded. Furthermore, the ongoing efforts to substitute traditional fuels with these environmentally friendly materials in the sintering process are introduced. The metallurgical properties of biocarbon are closely related to its chemical composition and physical characteristics, such as porosity, surface area, and internal structure. It has higher CO_2_ reactivity, lower electrical conductivity, higher SiO reactivity, and lower mechanical strength than conventional coke. Some of the drawbacks can be addressed through techniques such as densification, pyrolysis, carbonization, and agglomeration, effectively mitigating these limitations. Additionally, the current application situation on sintering has demonstrated that the substitution of specific coke amounts with biobased reductants in the ore agglomeration process can save energy. The incorporation of biocarbon in metallurgy is a feasible and potential way to reduce CO_2_ emissions, and this work deserves a valuable and significant endeavor.

## 1 Introduction

**Figure 1:**
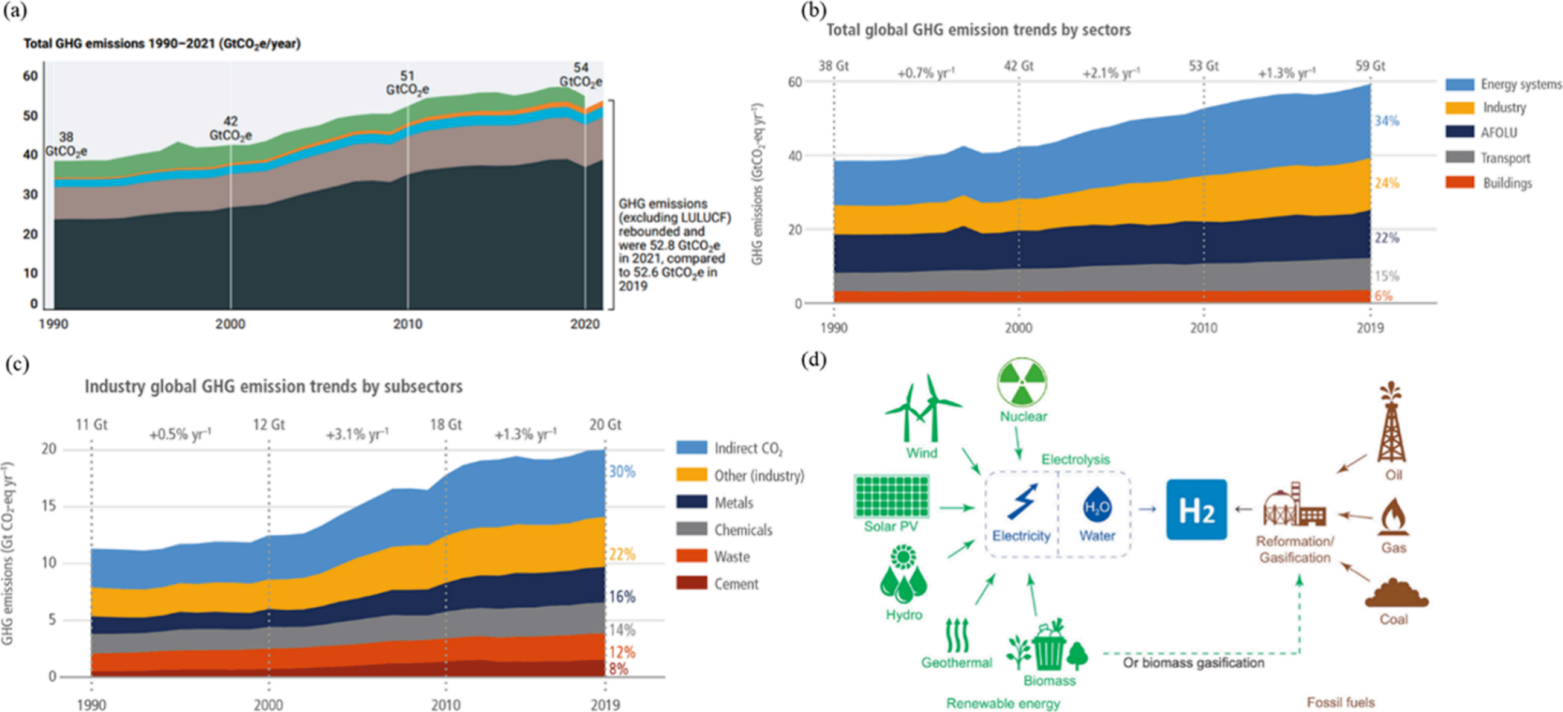
(a) The GHG emissions from 1990 to 2021. Reprinted with permission from ref (1). Copyright 2022 UNEP. (b) Total global GHG emission trends by sectors. Reprinted with permission from ref (2). Copyright 2022 IPCC. (c) Industry global GHG emission trends by subsectors. Reprinted with permission from ref (2). Copyright 2022 IPCC. (d) The recourses of hydrogen. Reprinted with permission from ref (12). Copyright 2021 KeAi.

Solid carbon plays an important role in metallurgical industries, as fuels and reductants. Rising quantities of fossil carbons are applied to meet the requirements of a developing industry. It has however caused damage to the environment because of the amount of greenhouse gas (GHG) emission, responsible for increasing the global temperature. Figure 1(a) shows the GHG emissions from 1990 to 2021.^1^ It can be seen that the rate of emissions growth has decreased over the past decades. The average annual growth rate was 1.1% between 2010 and 2019, compared to the period of 2009 to 2000 with the growth rate of 2.6%. However, the emissions were at the highest the last ten years, where the total annual emission is about 54 Gton CO_2_e (carbon dioxide equivalent).^1^ Among all contributors, the industry sector makes up the second-largest part when direct emissions are considered. When direct and indirect emissions are taken into account, it can be seen as the largest contributor. It is seen in Figure 1 (b) and (c) that, in 2019, approximately 34% of total GHG emissions came from the energy supply sector, 24% from industry, 22% from agriculture, forestry, and other land use (AFOLU).^2,3^ If emissions including electricity and heat production are attributed to the final energy, then 90% of these indirect emissions can be allocated to the industry and buildings sector.^2,3^

In the section of industry, metal productions cause considerable quantities of gas emissions due to fossil fuels, which is 16% of total industrial emissions.^2^ Iron and steel industry, as well as ferroalloy production, contributes to considerable amount of CO_2_. According to the statistics,^4^ 1.83 kg CO_2_ is emitted per kilogram steel. 1.04 to 1.19 kg, 1.4 to 6.9 kg, 2.5 to 4.8 kg CO_2_ will be emitted per 1 kg of ferromanganese (FeMn), silicomanganese (SiMn), and ferrosilicon (FeSi), respectively, as presented in Table 1. For comparison, the generic CO_2_ emissions from those alloys are equal to the mean CO_2_ emission of 11.8 million people.^5^

**Table 1:** Amount of CO_2_ Emission by Producing 1 kg of Products^4^

steel	FeMn	SiMn	FeSi
1.83 kg	1.04–1.15 kg	1.4–6.9 kg	2.5–4.8 kg

From the perspective of environmental protection, decarbonizing and reducing emissions in industrial field is an essential step. Some low carbon technologies have attracted increasing attention like electrolysis, prereduction of materials, hydrogen technology, and biocarbon replacing fossil fuels.^6^

Electrolysis is often used for producing reactive metals like sodium, potassium, lithium, magnesium and calcium, which are electrolyzed from their chlorides. Aluminum is obtained by electrolysis of molten cryolite (Na_3_AlF_6_).^7^ Manganese is electrodeposited from aqueous solutions through the electrolysis of Mn and an ammonium sulfate solution.^8^ However, electrolysis of the metal oxides is also difficult because of their poor solubility in the electrolyte melt, high temperature of operation, high affinity of the metal to oxygen, as well as low efficiency for multivalent metals.^9^

Hydrogen technology is the way to switch carbon to hydrogen, which can achieve the goal of carbon free production.^10^ Approximately 96% of the global hydrogen is produced from traditional fossil fuels, of which steam reforming of natural gas contributes to 48%, naphtha reforming accounts for 30%, and coal gasification is 18%.^11,12^ The recourses of hydrogen can be seen in Figure 1(d).^12^ It is reported that CO_2_ emission in iron–steel industry can be reduced by 78 to 95% when carbon is replaced by hydrogen, however, less than 10% of global hydrogen is produced for use in the metal industry.^13^ This is because most of metal production mainly depends on solid reductants currently, the original facilities need to replace totally if the hydrogen technique is going to be used. Additionally, the off gas will contain much unreacted hydrogen gas, which will also lead to carbon emissions unless the green hydrogen is used. Green hydrogen refers the hydrogen generated electrolysis of water by renewable energy or in other low carbon way. It is worth noting that the application of green hydrogen is limited today by the scarcity of efficient and cost-effective production technologies.^14^

Besides that, manganese raw materials which have been prereduced with H_2_ can be smelted in a submerged arc furnace to produce ferromanganese. It was reported that the energy consumption could be reduced from 3000 to 1600 kWh/t FeMn when prereduced pellets were used instead of raw ores in an electric furnace.^15,16^

Applying biocarbon in metallurgy as a green reductant is a promising way as it is a carbon neutral material,^17^ which undergoes a relatively short renewal time (less than 100 years) and involves the closed carbon cycle without additional CO_2_ emissions. The biocarbon in this paper refers to biomass from social life wastes^18−20^ and natural plants,^21,22^ charcoal (biocoal, biochar),^23,24^ as well as biooil.^25^ Metallurgical processes require large amounts of carbonaceous materials, and utilizing renewable biocarbon would reach two goals: (1) reduce CO_2_ emissions by substituting the fossil reductants and (2) increase utilization of abundant forest resource by producing higher value and energy products.^26^ For this purpose, The Federation of Norwegian Industries launched that the reasonable use of Norwegian biomass resources would target a reduction of 43% of the CO_2_ footprint of the metallurgical industry by 2030 compared to 2005 levels.^27^

Charcoal is one of the most common reductants among biocarbon in Mn-alloy and Si-alloy production. Some production processes for charcoal are discussed herein. The methods employed for its production are thermochemical technologies like carbonization, torrefaction, pyrolysis, and hydrothermal carbonization.^17^ Concerning both theory and the process, the first three methods are similar, while hydrothermal carbonization differs.

Pyrolysis is the thermal decomposition of biomass in an inert atmosphere. Throughout this process, the heating temperature, heating rate, and atmosphere are carefully controlled to achieve the desired composition and properties of charcoal.^17^ Carbonization, often referred to as slow pyrolysis, involves the conversion of wood to charcoal through gradual heating in an oven or kiln, typically at temperatures below 1200 °C, with a deliberately slow heating rate.^28^ Torrefaction is a method employed to enhance biomass properties. It involves the gradual heating of biomass in an inert atmosphere at temperatures ranging from 200 to 300 °C.^29^ However, using this method alone makes it challenging to significantly reduce sulfur and chlorine, thus converting them into the charcoal.^29^ Different pyrolysis temperatures, holding times, and types of biomass material affect the properties and compositions of charcoal. A pyrolysis temperature of 500 °C is insufficient to form a crystalline structure and enhance electrical conductivity. It is only when the temperature exceeds 800 °C that the micron size of crystallites and graphite structures begin to develop, as indicated by Surup’s research.^17^

Hydrothermal carbonization is an environmentally friendly decomposition process that transforms biomass into carbon-rich material through hydrolysis reactions with water, producing charcoal or hydrochar. The treatment temperature typically ranges from 450 to 520 K.^30^ Hydrothermal carbonization products with higher pore volume, surface area, and high adsorption capacity, make it suitable for use as an absorbent material. In contrast, pyrolyzed charcoal exhibits high carbon content and calorific value,^31^ making it possible to replace fossil fuel and fossil reductants.

In metallurgical processes, the common requirement for materials is low amounts of fines produced in the stage of transportation and alloy producing process, therefore a high mechanical strength is desired. At the same time, a high fixed carbon and density are in many cases needed for production.^32^ Biocarbon reductants’ mechanical strength, fixed carbon content, and density are relatively lower than metallurgical coke and coal,^33−37^ which can be seen in Table 2.

**Table 2:** Numerical Range of Properties of Different Carbon Reducing Agents^33−37^

	metallurgical coke	petrol coke	charcoal	coal	woodchips
fixed carbon %	87	84–90	65–87	60	12
volatile %	2	9–16	10–30	40	35
density kg/m^3^	500–550		180–350	800	250
mechanical strength kg/cm^2^	130–160		10–80	30	

Meanwhile, the metal production often needs to be carried out at high temperatures, so high temperature performances are also important evaluation indices of biobased reductants. The reducing agent should have high SiO-reactivity to meet the agreement of Si (silicon) or FeSi production, low CO_2_ reactivity, and low electrical conductivity for FeMn or SiMn production and low rate of air burn of anode requirements for Al production.^32,38^ Nevertheless, compared to the conventional reductant, the biobased agents usually possess relatively higher CO_2_ reactivity and electrical resistivity.^4,34^

This paper mainly focuses on the metallurgical properties of biocarbon involved in manganese- and silicon-alloy production. Through a comprehensive review of these properties, along with an investigation of challenges within the production process, better control of the metallurgical properties of biochar can be achieved in alignment with the Mn- and Si-alloy production processes. In this way, the target of producing high-quality alloys and reducing CO_2_ emissions can be achieved.

To sum up, environmental protection has put forward more strict restrictions on CO_2_ emissions. Therefore, industrial fields, especially metallurgical engineering, are in urgent need of implementation of decarbonization technology. The use of biocarbon as an alternative to conventional fuels is agreed to have great potential. But compared with the mature research of fossil fuels, biofuel is slightly inferior and needs to be explored in depth. In this paper, the methods of testing biobased materials at room temperature and metallurgical properties (CO_2_ reactivity, electrical resistivity, slag reactivity, SiO reactivity, mechanical strength) are summarized, the influence of characterizations of biocarbon on its performance behavior in furnace is discussed. In addition, the current situation of replacing conventional fuels with these green materials in sintering process is illustrated.

## 2 Metallurgical Properties of Biocarbons

### 2.1 CO_2_ Reactivity

The carbon materials in FeMn production will react with CO_2_ to CO, also known as the Boudouard reaction.^39,40^ The reaction equation is shown as eq 1.
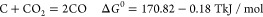
1

The Boudouard reaction is a result of the CO_2_ from the prereduction of manganese oxides in a submerged arc furnace. The Boudouard reaction is endothermic and hence absorbing heat, causing consumption of power.^4^ In addition, Boudouard reaction is undesirable from the prospective of increasing greenhouse gas emissions as the emitted CO will convert to CO_2_ at a later stage. It has been reported that almost 500 000 tonnes of CO_2_ emitted annually in manganese alloys production are involved in this reaction, corresponding to approximately 30% of the emissions per year.^41^ In this section, the term of “reactivity” is not the same as the meaning in kinetics, it is used for describing the whole process.

Studying the reactivity toward CO_2_ is important prior to the application of bioreductants. To clarify the gasification behavior of biobased reductants, the thermogravimetric analyzer (TGA) is often employed. The standardized measurement for coke reactivity index (CRI) is one of the methods that can use TGA for assessing biocarbon reactivity. The concept of CRI has been proposed by both ISO and ASTM standard systems to test the CO_2_ reactivity of coke, whose formula can be seen in eq 2.^42,43^ In this equation, *W*_i_, *W*_f_ represent the weight of sample at the beginning and at the end of gasification.
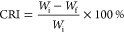
2

Table 3 provides a summary of various methods used to determine the CO_2_ reactivity of natural carbons and traditional coke, along with the corresponding conclusions. As can be seen in the Table 3, the experimental temperature, atmosphere, and duration of the experiments differ based on the specific research goals. Despite these variations, a consistent finding emerges that charcoal exhibits a higher CO_2_ reactivity compared to cokes. Additionally, the CO_2_ reactivities of biocarbons sourced from various woody biomasses were assessed by using both the standardized furnace for testing Coke Reactivity Index (CRI) and a thermogravimetric analyzer (TGA).^45^ The outcomes indicated that despite quantitatively differing results between the two experimental approaches, the final conclusions remained consistent.

**Table 3:** Experimental Parameters of Testing Reactivity towards CO_2_ and Reactivity Results

objective	test method	study temperature	atmosphere	reactivity order	ref
1. industrial charcoal	TGA	1070 °C	50% CO_2_ + 50% CO	raw charcoal > densified charcoal > coke	(4)
2. metallurgical coke					
3. densified charcoal					
1. coke	TGA	1000 °C	100% CO_2_	coke + 5% charcoal > coke + 2% charcoal ≈ coke + 3% charcoal	(44)
2. coke + 2% charcoal					
3. coke + 3% charcoal					
4. coke + 5% charcoal					
1. carbonized spruce wood residue	1. TGA	850 °C	100% CO_2_	spruce forest residue > spruce wood residue > birch forest residue > birch wood residue > industrial charcoal > coke	(45)
2. carbonized spruce forest residue					
3. carbonized birch wood residue					
4. carbonized birch forest residue	2. standard test for CRI				
5. industrial charcoal					
6. coke					
1. anthracite	1. TGA	1100 °C	100% CO_2_	charcoal 2 > charcoal 1 > lemon hydrochar > anthracite	(46)
2. charcoal 1 (pyrolyzed at 400–500 °C for 24 h)					
3. charcoal 2 (pyrolyzed at 700–750 °C for 5–10 h)					
4. lemon hydrochar (produced by hydrothermal carbonization)	2. standard test for CRI				

**Figure 2:**
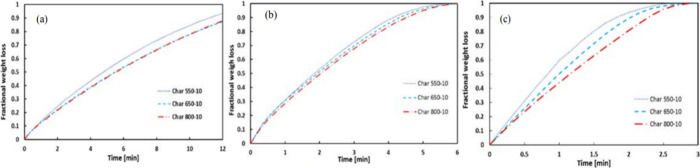
Weight loss as function of time for CO_2_ gasification of spruce wood biocarbon samples at temperatures of (a) 850 °C, (b) 900 °C, and (c) 950 °C. Reprinted with permission from ref (47). Copyright 2017 Elsevier.

Wang et al. investigated the CO_2_ reactivities of spruce wood biocarbons, which were produced at different temperatures (550 °C, 650 °C, and 800 °C) and same holding time (10 min).^47^ As evidenced by Figure 2, the CO_2_ gasification conversion of biocarbon produced at lowest temperature (550 °C) is faster than the two samples produced at higher temperatures (650 °C and 800 °C). Increasing gasification temperature is also seen to increase the CO_2_ reactivity of the whole gasification process.

According to previous studies, charcoal’s CO_2_ reactivity is much higher than metallurgical coke.^4,43−46^ It is believed that the high porosity and surface area of biocarbon are the contributing factors.^4,48,49^ Kaffash et al.^4^ observed that the CO_2_ reactivity of all charcoals decreased during the process of increased densification. This decline in reactivity was likely attributed to the reduction in porosity caused by densification. It is in agreement with the results of Barbieri and his team, who studied the combustibility and reactivity of coal blends and charcoal fines.^48^ In their study, charcoal has a higher CO_2_ reactivity than three typical PCI (Pulverized Coal Injection) coals for the high surface area of charcoal. The experimental data are listed in Table 4. As observed in Table 4, charcoal exhibits the most substantial specific surface area according to BET measurements. This alignment with the highest R_50%_ and the shortest t_50%_.

**Table 4:** CO_2_ Reactivity Test Results and Specific Surface Area of Carbon Reductants^48^

sample	*R*_50%_^a^ (min^–1^)	*t*_50%_^b^ (min)	*S*_BET_^c^ (m^2^/g)
charcoal	0.0592	7.8	172.3
coal A	0.0165	48.7	14.0
coal B	0.0175	23.1	2.9
coal C	0.0130	26.2	1.5

a *R*_50%_: Reactivities to CO_2_ of chars at 50% conversion.

b *t*_50%_: Time to achieve 50% conversion.

c *S*_BET_: Specific surface area determined on N_2_ isotherms by the BET method.

**Figure 3:**
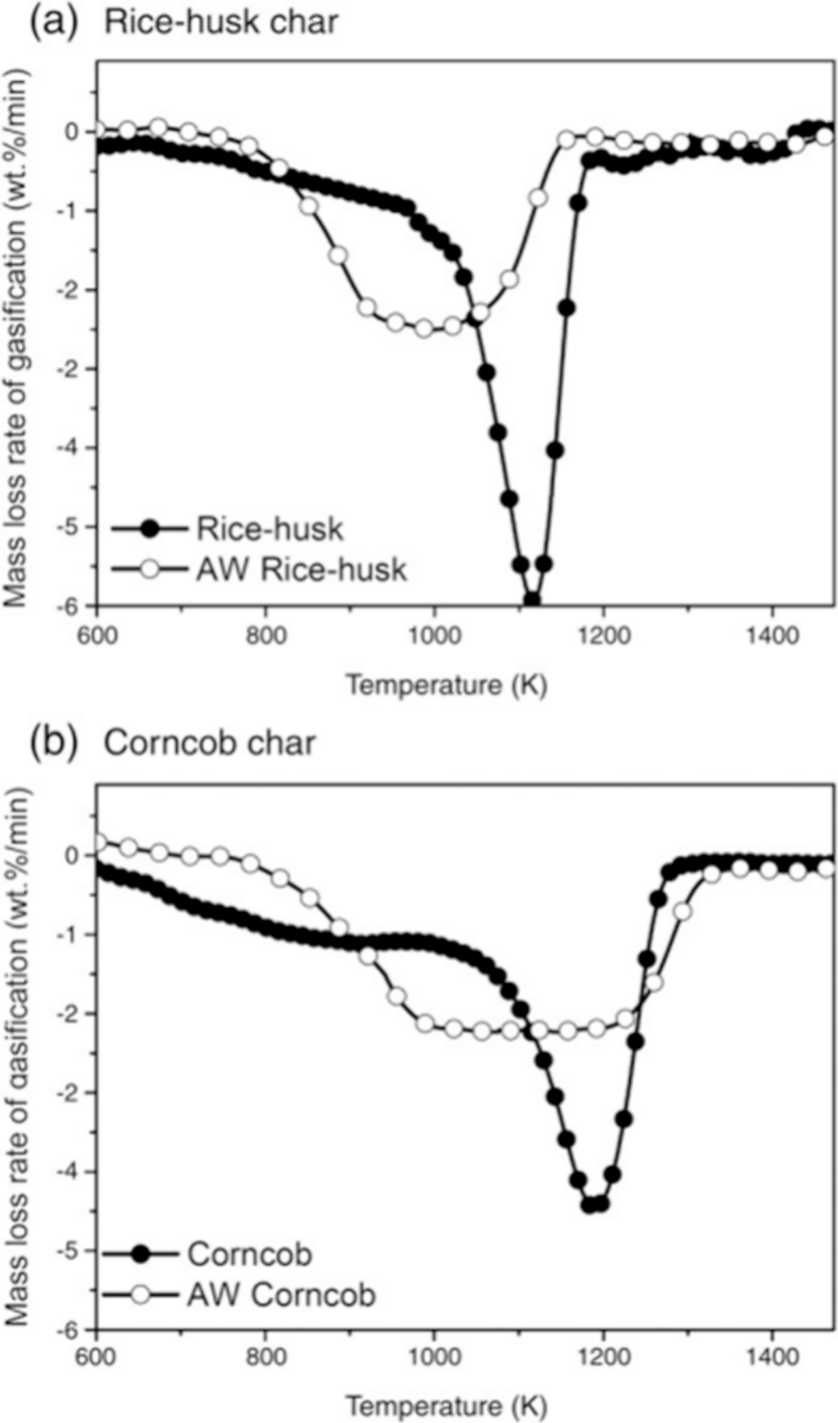
DTG curves during gasification of unwashed and acid-washed biomass char in CO_2_ atmosphere. Reprinted with permission from ref (51). Copyright 2010 Elsevier.

Ash content also plays a vital role in the reactivity with carbon dioxide. Scholars emphasize that the presence of metals within the ash in particular, influences the gas reaction behavior of bioreductants.^50,51^ Lv et al.^51^ conducted research into the impact of alkali and alkaline earth metallic (AAEM) species on the CO_2_ reactivity of biochar. In their study, acid-washed biomass (AW biomass) without AAEM species had a lower reaction rate compared to natural biomass possessing higher AAEM species content. The results are depicted in Figure 3. It is apparent that the acid-washed samples exhibit lower peak values compared to the unwashed samples, and the gasification temperature decreases. The authors explained the reason that the AAEM species existing in biomass catalyze the gasification reaction resulting in increasing char reactivity.

**Figure 4:**
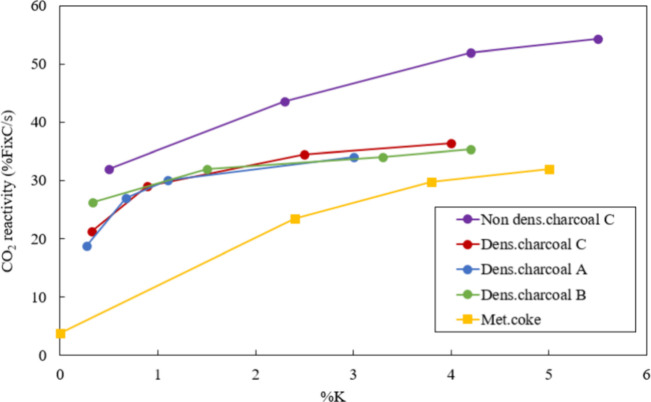
CO_2_ reactivity versus potassium concentration. Reprinted with permission from ref (52). Copyright 2022 Springer Nature.

Kaffash et al. also explored the effect of K element on the CO_2_ reactivity of carbonous material.^52^Figure 4 showed that the reaction rate rises with potassium concentration. Regarding densified charcoals, the reaction rate reaches a constant value at a concentration of around 1 wt % K, but for nondensified charcoal and metallurgical coke, the particular concentration is about 4 wt % K. This is in agreement with results from Alam et al.^53^ and Rao et al.,^54^ where the coke which was impregnated by potassium compounds (KCN, KOH, or K_2_CO_3_) showed a higher Boudouard reaction rate at the temperature range from 800 to 1200 °C.^55^

### 2.2 Electrical Resistivity

**Figure 5:**
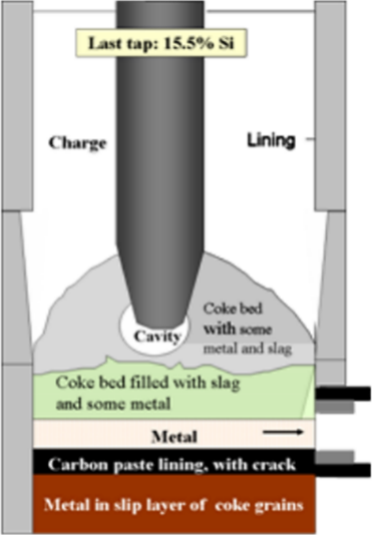
Diagram of coke bed in a pilot scale submerged arc furnace. Reprinted with permission from ref (34). Copyright 2007 International Committee on Ferro-Alloys (ICFA).

The production of ferromanganese (FeMn) typically takes place in a submerged arc furnace (SAF). The coke bed consists of coke, slag, and metal. Gas also flows through the bed, which can be seen in Figure 5.^34^ In the smelting process, the current passes through the coke bed, making its electrical resistivity a crucial factor. In the other respect, the utilization of carbonized charcoal as a consumable anode in the carbon fuel cell, which is a battery usually using a consumable carbon anode to generate power, has been studied by scholars.^56−58^ The electrical property of carbonized charcoal is required to fully explore the development of biocarbon fuel cells. Therefore, the electrical properties of biocarbon deserve in-depth study.

**Figure 6:**
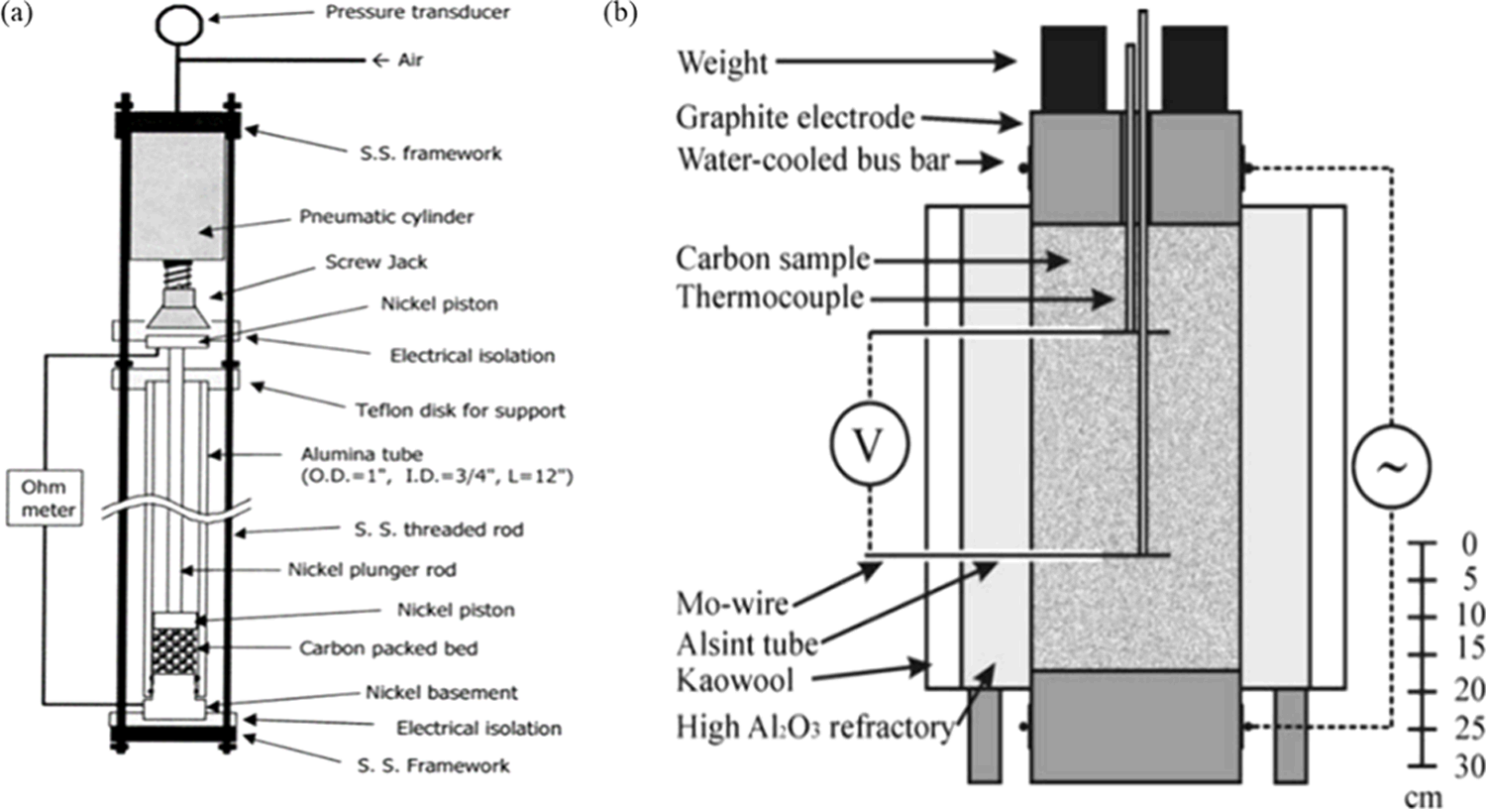
Schematic diagram of electrical resistivity measuring equipment. (a): Two probe method. Reprinted with permission from ref (58). Copyright 2003 American Chemical Society. (b) Four-electrode method at high temperature. Reprinted with permission from ref (59). Copyright 2008 Springer Nature.

The electrical property of materials is often measured by s two or four probes technique. The schematic diagram of the two and four probe apparatus used to measure resistivity can be seen in Figure 6.^58,59^ For the two-probe method, as illustrated in Figure 6(a), a setup involves nickel electrodes positioned at the top and bottom of a 1.9 cm diameter packed bed, which is enclosed within an alumina tube. This configuration allows for the measurement of the electrical resistance of the bed. The resistivity ρ (Ωcm) of the bed can be calculated using the equation:  where *R* is the measured resistance of the bed in ohms (Ω), *A* is the cross-sectional area of the bed in square centimeters (cm^2^), and *l* is the distance between the two probes in centimeters (cm).^58^Figure 6(b) shows a four-probe apparatus. The setup involves a castable high-alumina refractory cylinder built around both an upper and a lower 304.8 mm standard graphite electrode. Water-cooled copper bus bars are used to connect the power supply to the top and bottom graphite electrodes. In this method, the following equation is used to calculate the bulk resistivity ρ of the materials: , where *U* is the measured voltage drop, *I* means the measured current, *A* is the cross-sectional area of the coke bed, and *h* represents the distance between the measuring points.^59^

The electrical resistivities of various biocarbon materials are examined,^58−60^ and their results are listed in Table 5. It is noticeable that the electrical resistivity of charcoal is usually between metallurgical coke and petroleum coke, where the metallurgical coke exhibits lower electrical resistivity. The electrical property of charcoal is primarily influenced by the initial material. Additionally, the treatment temperature has a significant impact on the electrical resistivity.

**Table 5:** Summary of the Resistivity of Different Types of Biocarbon and Conventional Coke

origins	treatment temperature (°C)	examine temperature ( °C)	electrical resistivity (Ωm)	method
Macadamia nutshell^58^	650	20	6.6 × 10^1^	two-probes technique
Macadamia nutshell^58^	750	20	2.9 × 10^–1^	two-probes technique
Macadamia nutshell^58^	850	20	5.8 × 10^–3^	two-probes technique
Macadamia nutshell^58^	950	20	1.1 × 10^–3^	two-probes technique
Macadamia nutshell^58^	1050	20	5.9 × 10^–3^	two-probes technique
Kukui nutshell^58^	950	20	1.8 × 10^–3^	two-probes technique
Coconut shell^58^	950	20	1.8 × 10^–3^	two-probes technique
Leucaena wood^58^	950	20	1.6 × 10^–3^	two-probes technique
Activated carbon^58^	950	20	1.1 × 10^–3^	two-probes technique
Metallurgical coke^89^		1550	3.9 – 10.2 × 10^–3^	four-probes technique
Petroleum coke^59^		1550	8.13 – 13.3 × 10^–3^	four-probes technique
Brazilian charcoal^59^		1500	8.11 × 10^–3^	four-probes technique
Indonesian charcoal^59^		1500	9.11 × 10^–3^	four-probes technique
hydrothermal carbonized olive^60^	200	20	0.012–0.020	four-probes technique
hydrothermal carbonized olive^60^	220	20	0.013–0.030	four-probes technique
hydrothermal carbonized olive^60^	240	20	0.006–0.012	four-probes technique

**Figure 7:**
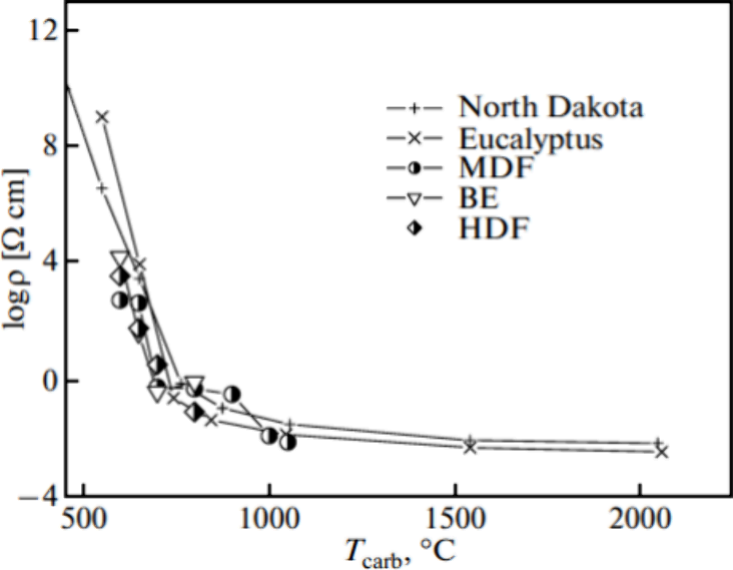
Dependences of the electrical resistivity of different carbonized woods at *T* = 300 K on the carbonization temperature. Reprinted with permission from ref (64). Copyright 2011 Springer Nature.

The conduction occurs because of charge carrier tunneling between conducting grains.^61^ Moreover, the electrical conductivity of carbonaceous material relays on the intraparticle resistance and the interparticle contacted resistance, which are affected by temperature.^62,63^ Consequently, it is accepted that the electrical property of biocarbon is closely related to its structure and therefore the pretreated temperature. Popov et al.^64^ explored the relationship between carbonization temperature and electrical resistivity. As seen in Figure 7, all the carbonized woods adopted in this experiment have a similar trend, which shows that the electrical resistivity declined with increasing of carbonization temperature during the temperature range of 500 °C to 1000 °C, and beyond this range it is close to constant. They concluded that, as the carbonization temperature increases, the structure is ordered inhomogeneously over the sample volume, which results in the variation of the electrical resistivity. Parfen’eva and his team also found that the resistivity increases with decreasing temperature by investigating the eucalyptus and white pine wood, while the correlation is not high.^65,66^

Chang et al. concluded that the electrical conductivity of biocarbon is influenced by two major factors: surface area and the degree of graphitization.^67^ The larger surface area and superior porosity of biocarbon contribute to its better electrochemical properties, enabling efficient ions transfer, high charge storage. The higher graphitization degree is advantageous to the fast electron transfer. This was also supported by Zhao et al.’s study.^68^ The activated carbon was treated at 5 GPa and up to 1600 °C to investigate the effect of pressure and temperature on the graphitization process. The results indicated that the graphitization time is 1200 °C. The structure of activated carbon transited from nongraphitization to near-graphitization to graphitization with the increase in temperature. The electrical property is highly consistent with the graphitization process.

Some studies also mention the effect of oxygen content when it comes to electrical conductivity of carbon material.^69^ The biocarbon obtained by almond shells and hulls at 700 °C showed an overall improvement in conductivity compared to the products generated at 300 and 500 °C,^69^ which is associated with the lower oxygen content of the biocarbon gained by 700 °C. Ana et al.^70^ explained this phenomenon further. The surface of the particles is made up by carbon and oxygen, when the materials experienced isopropanol and blank annealing, the oxygen concentration decreased, which increase the material’s electrical conductivity.

**Figure 8:**
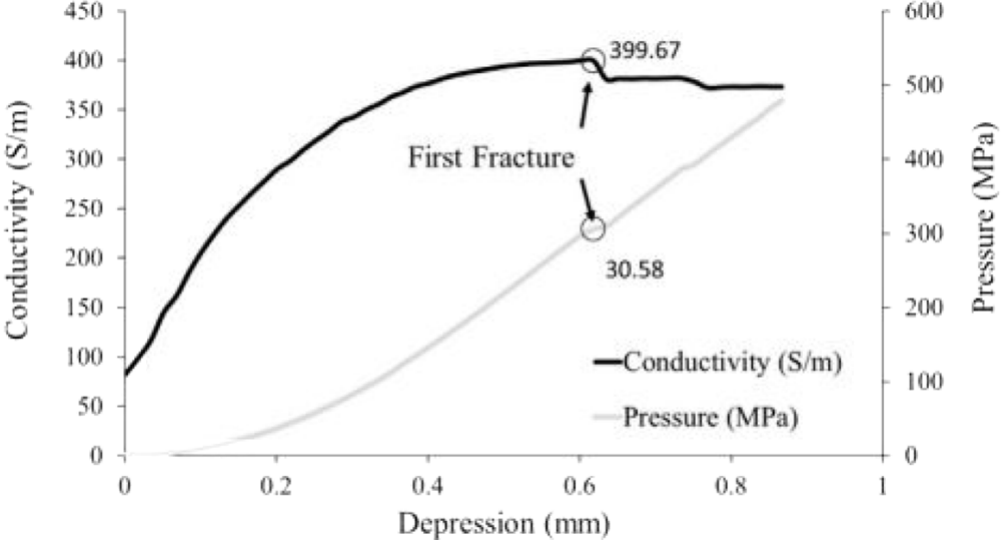
Electrical conductivity versus compression of sugar maple biochar. Reprinted with permission from ref (71). Copyright 2017 Elsevier.

The relationship between electrical conductivity and compression of biochar was studied by Gabhi et al.^71^ In their research, the electrical conductivity of biochar was found to rise with compressive loading until the internal fractures were generated, which is not beneficial to the conductivity. They explained that this phenomenon corresponds to “elastic behavior of electric conductivity of biochar” and this is also consistent with the anticipated dependence between conductivity and structure. Figure 8 shows the variation of electrical conductivity with increasing depression.

**Figure 9:**
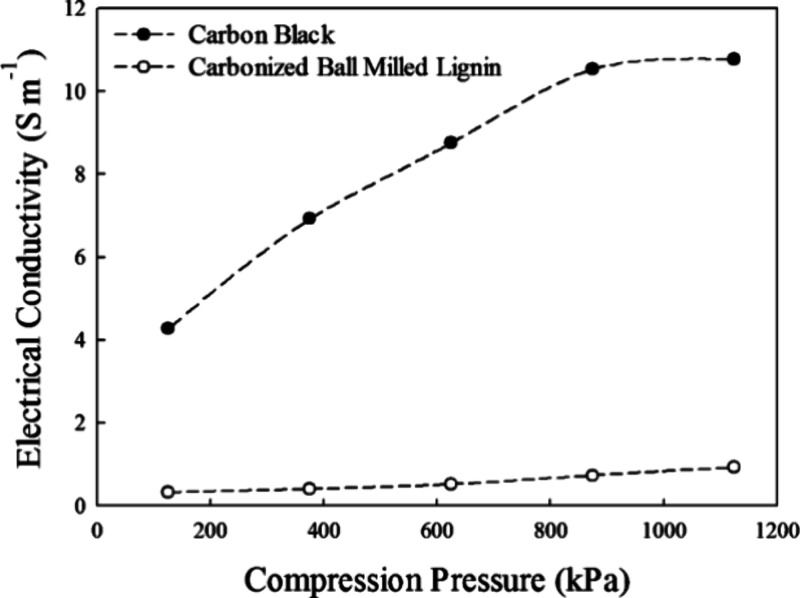
Electrical conductivity versus compression pressure of carbon black and carbonized ball milled lignin. Reprinted with permission from ref (21). Copyright 2014 American Chemical Society.

Snowdon et al. measured the electrical conductivity of carbon black and carbonized ball milled lignin.^21^ During the experiment, a pressure range of 125 kPa to 1.12 MPa was applied by adding additional weight on top of the upper piston. This pressure range was carefully selected to ensure that the particles did not crack, while still enabling good electrical contact between the powder and pistons. The result was represented in Figure 9, which shows that carbon black became more conductive with increasing compression pressure.^21^ In comparison, carbonized lignin showed a slight improvement. The difference, which existed in the electrical conductivity of the carbonized lignin and the carbon black, may come from the ball milled carbonized lignin containing a higher content of residual oxygen, while most of the oxygen species were reduced in carbon black. This finding aligns with the understanding that surface elements other than carbon tend to decrease the overall conductivity of the material.^72^

### 2.3 Slag Reactivity

If biocarbon is used as a reducing agent in metal production, then it will be involved in the reduction reaction with slag. In the production of FeMn and SiMn, the most important reactions are between slag and carbon as follows:^36^
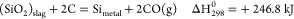
3
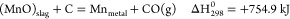
4

**Figure 10:**
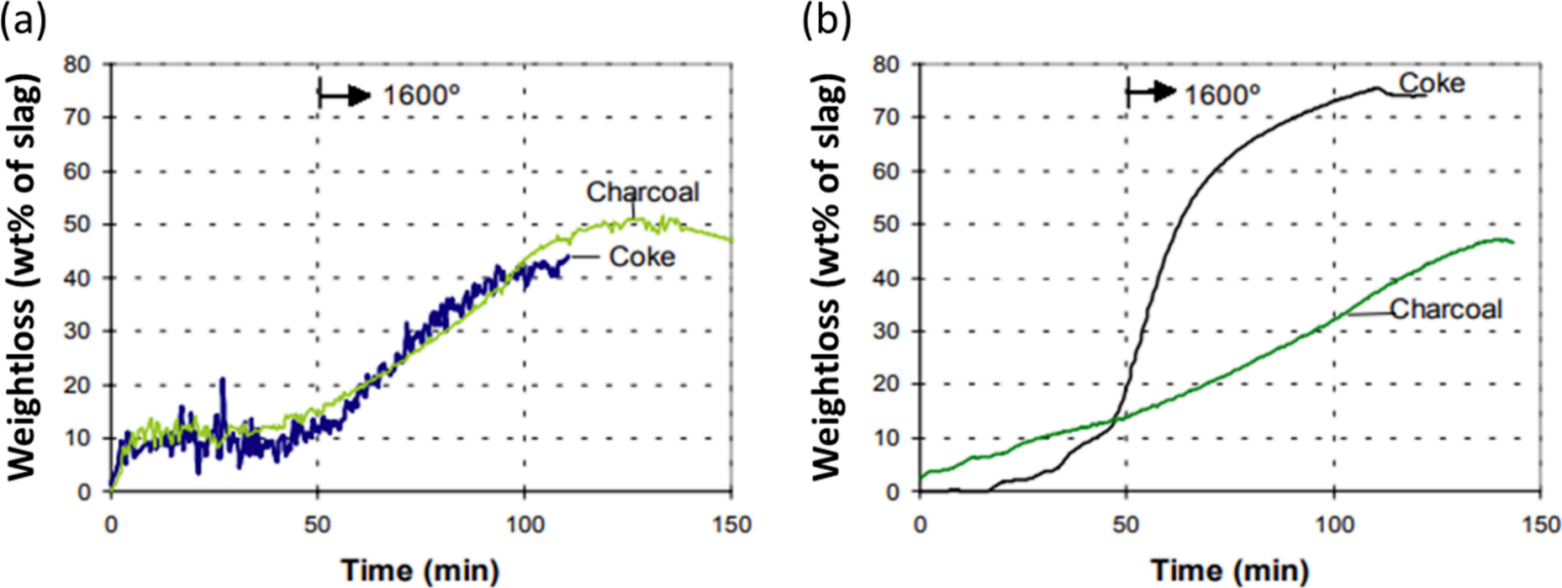
Slag-reactivity at 1600 °C for charcoal and coke; (a) coke precalcined at 1600 °C. (b) Coke precalcined at 1200 °C. Reprinted with permission from ref (74). Copyright 2004 International Committee on Ferro-Alloys (ICFA).

Various methods have been used to measure the slag reactivity. Thermogravimetry at 1600 °C was employed to test slag reactivity by Solheim et al.^73^ The synthetic slag was filled to the rim into the crucibles made of the given carbon material, and the results were represented by weight loss of mixed materials. In their study, it was found that charcoal exhibited an equally good slag reactivity compared to coke, as the Si content of all the products reached 20%. The differences are that if the coke had been calcined at 1600 °C for 3 h, the weight loss is identical to that using charcoal. The rate of reaction was faster for coke if a lower calcination temperature (1200 °C) was used, which can be seen in Figure 10.^74^ It is mainly because coke still has the higher ash content than charcoal after experiencing relatively lower calcined temperature. The excess ash would react with carbon which has been dissolved in slag during the experiment.

In the study conducted by Gaal et al.,^75^ they investigated the reduction behavior of industrial slag (MnO and SiO_2_ containing slags) using various carbon materials, including industrial coke, anthracite, and eucalyptus charcoal. The experimental setup involved heating 500 g of industrial slag in a graphite crucible to 1600 °C and holding it for 20 min before adding 100 g of one of the carbon reductants to the crucible. After the tests, the crucible contents were quenched in a graphite mold, and the reductants were separated, weighed, and analyzed. The results showed that charcoal exhibited good reduction kinetics, as it reduced more of the slag compared to both coke and anthracite. Additionally, charcoal produced more metal per unit of carbon and had a higher concentration of Si in the metal.

**Figure 11:**
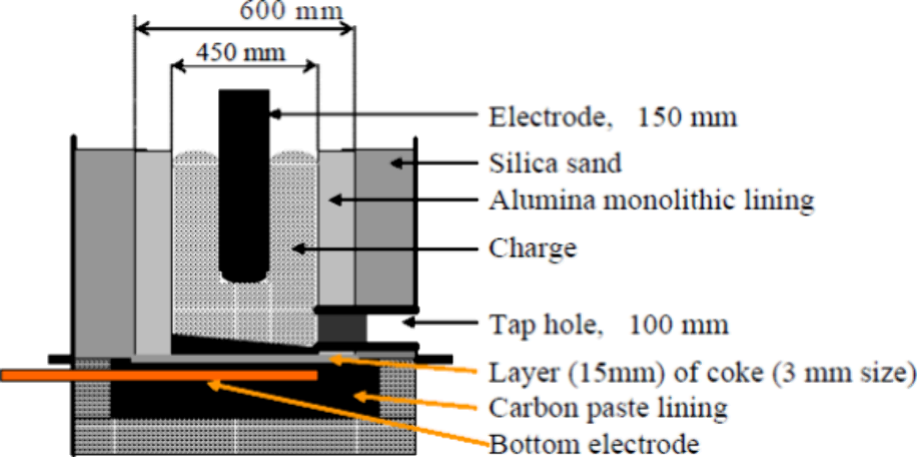
Sketch of the setup used by Monsen et al. Reprinted with permission from ref (74). Copyright 2004 International Committee on Ferro-Alloys (ICFA).

In the work conducted by Monsen et al.,^74^ five pilot scale experiments were carried to produce silicomanganese. The experimental setup employed a 150 kW single electrode furnace, and Figure 11 provides a schematic drawing of the setup. The main objective of these experiments was to investigate the use of three different reductants: industrial coke, reactive coke, and charcoal, for producing silicomanganese with a target silicon content of 18%. The researchers aimed to study any differences in the coke beds when different reductants were utilized. The raw materials used in the experiments included manganese ores (35 kg), HC FeMn slag (15 kg), and quartz. The charging of the furnace started with a coke bed weighing 3 or 5 kg, as detailed in Table 6.

**Table 6:** Charge Composition Used in the Five Pilot Scale Experiments^74^

run no	coke bed (kg)	charged materials	moisture (%)	charge 1 (kg)	charge 2 (kg)	charge 3 (kg)	charge 4–11 (kg)	
1	5	industrial coke	13	11.5	12	13	13.5	
		quartz		0	6.5	11	13	
2	3	industrial coke	2	10	10.5	11.5	12	
		quartz		0	6.5	11	13	
3	*5*	charcoal E1	5	11.5	12	13	13.5	
		quartz		0	6.5	11	13	
4	5	charcoal E2	7	11.5	11.5	12.5	13.0	12.5
		quartz		0	6.5	11	13.3	12.5
5	3	charcoal E1	7	5.5	6	6.3	6.6	
		industrial coke	0	4.8	5.2	5.5	5.7	
		quartz		0	6.5	11	13.3	

The findings revealed the following: Charcoal yielded higher metal production compared to coke as a reductant. However, the metal produced using charcoal had the lowest silicon content. Reactive coke resulted in intermediate metal production and silicon content in the produced metal, falling between the values observed for charcoal and industrial coke. Industrial coke exhibited the lowest metal production, but the metal produced had the highest silicon content. Additionally, the use of charcoal as a reductant increased the total resistance in the coke bed, and it led to the highest slag production during the process.

For the further exploration, Monsen et al.^34^ conducted a pilot test with metallurgical coke, reactive coke, and charcoal as reducing agents in an induction furnace. The researchers maintained the same amounts of HC FeMn slag, quartz, and ore on a dry basis as run no.5, as listed in Table 7. The differences are the starting coke bed was set to 5 kg, and the ore weight was increased to 38.8 kg in run no. 6. The metal analysis of run no. 6 revealed that it had a higher silicon content in the produced metal compared to the previous runs. However, it was observed that there was a lower metal production in this run. This outcome indicates that the choice of reductant and other process parameters can influence the final metal composition and production.

**Table 7:** Charge Composition Used in Monsen et al.’s Study^34^

run no	coke bed (kg)	charged materials	moisture (%)	charge 1 (kg)	charge 2 (kg)	charge 3 (kg)	charge 4–11 (kg)
5	3	charcoal E1	7	5.5	6	6.3	6.6
		industrial coke	0	4.8	5.2	5.5	5.7
		quartz		0	6.5	11	13.3
6	5	charcoal E1	7	5.5	6	6.3	6.7
		industrial coke	8	5.1	5.6	6.0	6.3
		quartz		0	6.5	11	13.3

**Figure 12:**
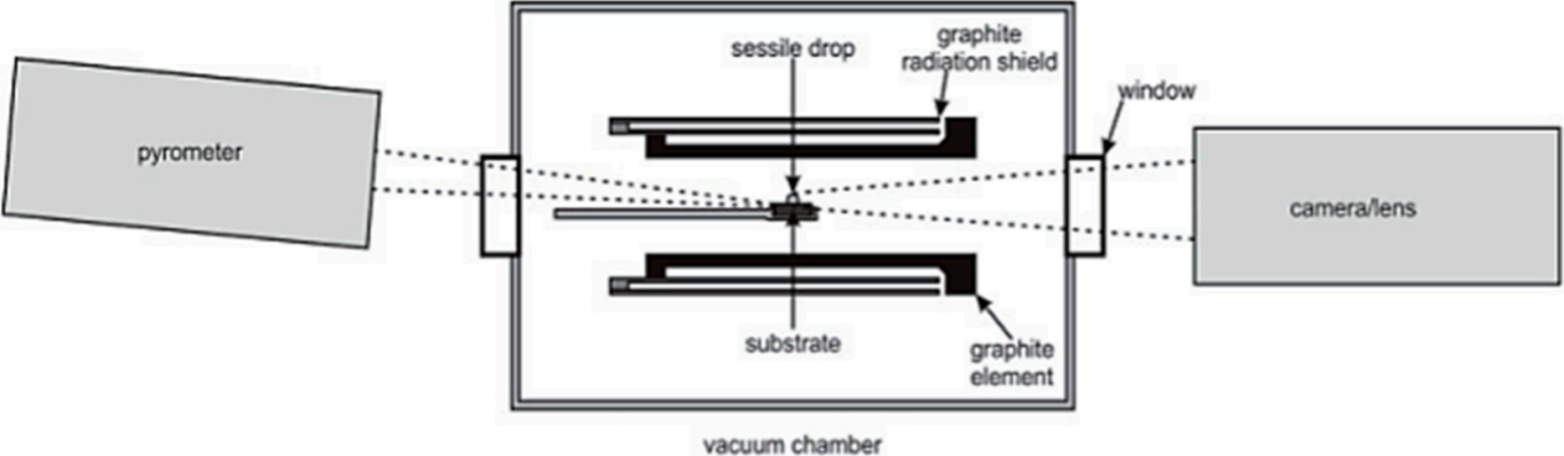
Schematic diagram of the sessile drop setup. Reprinted with permission from ref (76). Copyright 2010 International Committee on Ferro-Alloys (ICFA).

In manganese alloy production, manganese oxides can involve reduction behavior from the liquid slag by solid carbonaceous reductant. The interactions between slag drop and solid carbon influence the Mn yield in the metal. The approach of sessile drop wettability is often used to investigate the contact between a solid substrate and a liquid phase. At the same time, it is considered an optimal approach because it allows for the preparation of a more homogeneous substrate, which in turn is more representative of the selected carbon material.^76^ An example of a sessile drop setup is exhibited in Figure 12. The carbon substrate is placed in the graphite sample holder and the slag sample is put on the carbon surface.

**Figure 13:**
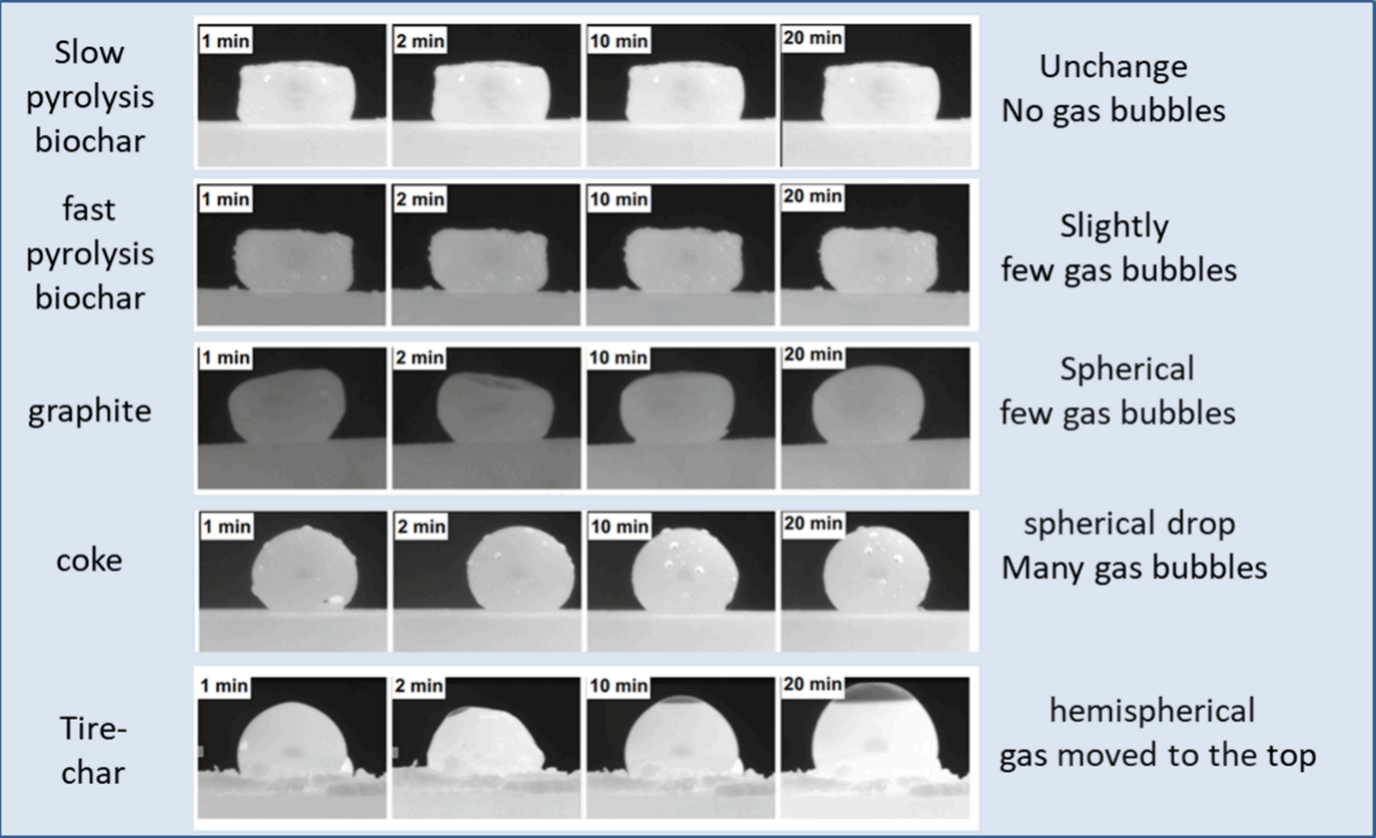
Slag and carbon interfacial phenomena with different carbonaceous materials. Reprinted with permission from ref (77). Copyright 2019 Springer Nature.

Huang et al. studied the interactions of carbon materials with slag in the sessile drop setup.^77^ The degree of interfacial reactions of slag and carbon were ranked based on visual observations of the generated gas bubbles, considering factors such as quantity and volume, as well as the movement and shape evolution of the slag. Figure 13 showed various interfacial phenomena between slag and different carbonaceous materials. It can be observed that biochars (both slow pyrolysis biochar and fast pyrolysis biochar) exhibited the least interactive behavior among the carbonaceous materials, however, tire-char had the most intense interfacial reaction.

**Figure 14:**
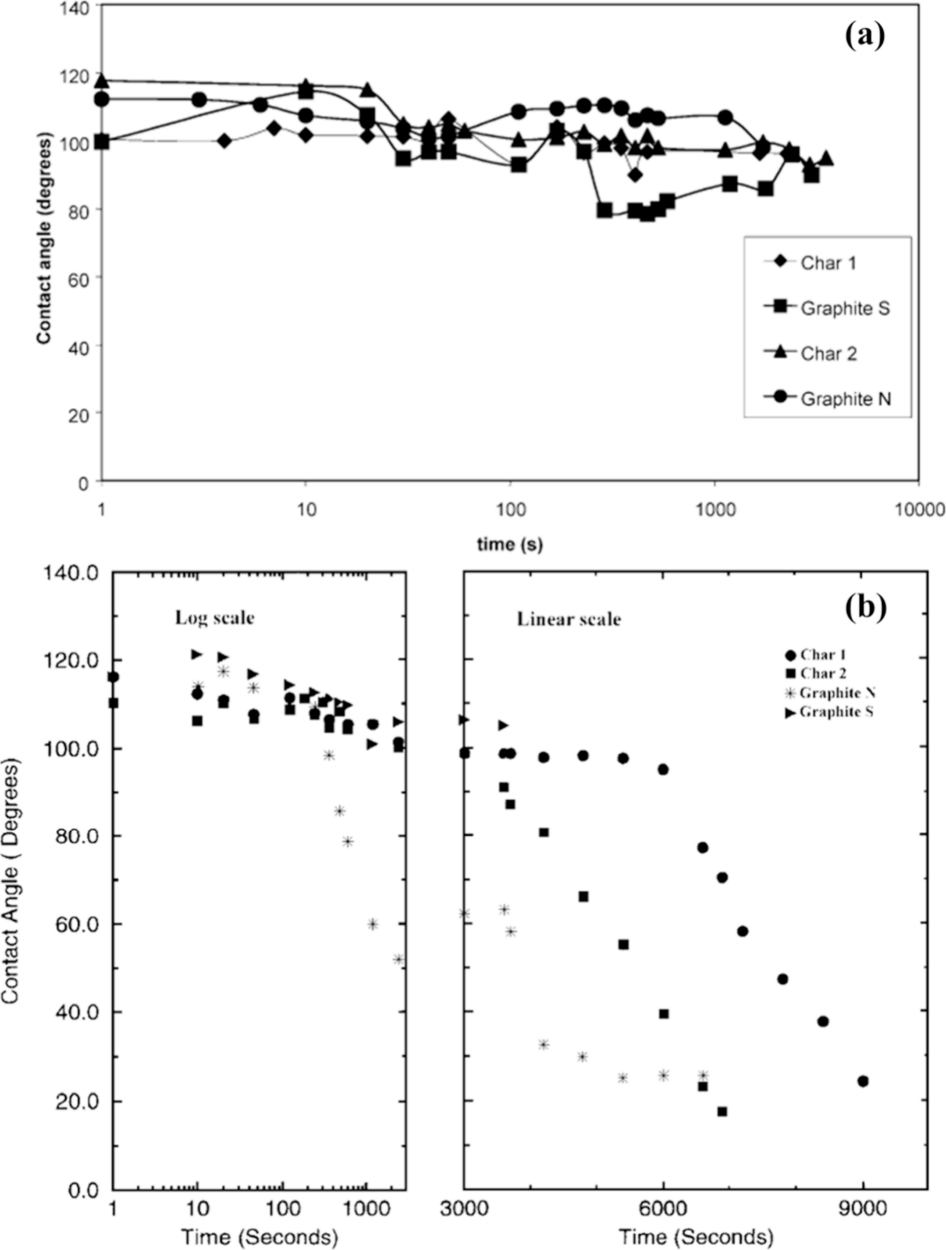
Variation of contact angle as a function of time for various carbonaceous materials with slag 1 and slag 2: (a) slag 1, (b) slag 2. Reprinted with permission from ref (78). Copyright 2003 The Iron and Steel Institute of Japan.

Mehta and Sahajwalla^78,79^ conducted an analysis of the wetting behaviors of coal-char and slag interactions by using four carbonaceous materials (synthetic graphite, natural graphite, and two chars) and two different slags. One slag had higher FeO content (slag 1), while another was with higher SiO_2_ content (slag 1). The variations of contact angle of different interactions as a function of time were shown as Figure 14. It can be noticed that all four carbonaceous materials exhibited nonwetting behavior with limited changes over time when reacting with slag 1. As for slag 2, synthetic graphite continued to retain nonwetting behavior, while natural graphite and the two chars exhibited dynamic wetting after a certain period. The dynamic wetting started after 3000 s for natural graphite, and 7000 s for char 1 and 5000 s for char 2, respectively. They explained that these phenomena can be attributed to the deposition of products at the interface. For slag 1, a large amount of reduced Fe deposition appeared to dominate the wettability of the slag. For slag 2, due to the carbon depletion and deposition of SiC, the interfacial reaction slowed down, and the supply of silica began to exceed the consumption, which led to the continuous reduction of interfacial tension.

Surup et al.^80^ analyzed the reactions behaviors of graphite, charcoal, coal char, and metallurgical coke with FeMn slags. They found that charcoal, coal char, and metallurgical coke presented a nonwetting behavior at the temperature lower than 1650 °C. Applying pressure to the burden did not significantly enhance the flow into the charcoal or coal char matrix. However, small amounts of slag were observed to be recovered within the metallurgical coke bed. Sessile drop experiments confirmed that at temperatures above 1600 °C, the FeMn slag penetrated the outer layers of the carbon particles in both charcoal and metallurgical coke. In the research of Safarian and Kolbeinsen,^81^ it was found that the graphite substrates are not wetted by the high-carbon ferromanganese slag at 1450 °C, 1500 °C, and 1600 °C. Additionally, the reduction rate of MnO rises with the surface increase in roughness, which suggested that a rougher surface facilitates the reduction reaction. The surface area of graphite has a minor effect on the reaction kinetics, while an increase in the porosity and pore size of graphite leads to a decrease in the rate of slag reduction. These two findings both proved that the porosity or pore size have certain influence on the carbon-slag reactions.

Rahman et al. analyzed the reactivity of EAF slag droplet (34.8% Fe_2_O_3_) slag with metallurgical coke and natural graphite.^82^ The size of the slag droplet showed small but continuous fluctuations in size associated with the generation and subsequent release of gas for coke. However, for the natural graphite substrate, the size of the slag droplet showed a significant increase with time compared to its initial size. Moreover, they believed that the dominant difference is the disparity in ash content, with 2% in graphite and 18% in metallurgical coke. Slag is generally expected to exhibit better wetting behavior with ash oxides compared to carbon.^83^ For the metallurgical coke, which contains 18% ash, CO bubbles formed along the interface of carbon and slag would easily grow vertically due to high contact angle with ash particles, which can form a 2D interconnected network along the surface. As for the graphite with 2% ash content, the bubbles grew with a low contact angle, covering the entire carbon surface as 2% ash is not able to form a 2D surface network. It is also explored that increasing silica content in ash can lower the surface tension of slag because it is a very surface-active part in slags. The smaller surface tension of slag will lead to the reduction in the size of gas bubbles, and the weakness of ability to hold gas. Apart from silica, alumina is also significant impurity oxide in coke ash, the reaction between alumina and melt showed a very undesirable wetting behavior, which can also influence the interaction between coke and slag.^84^

**Figure 15:**
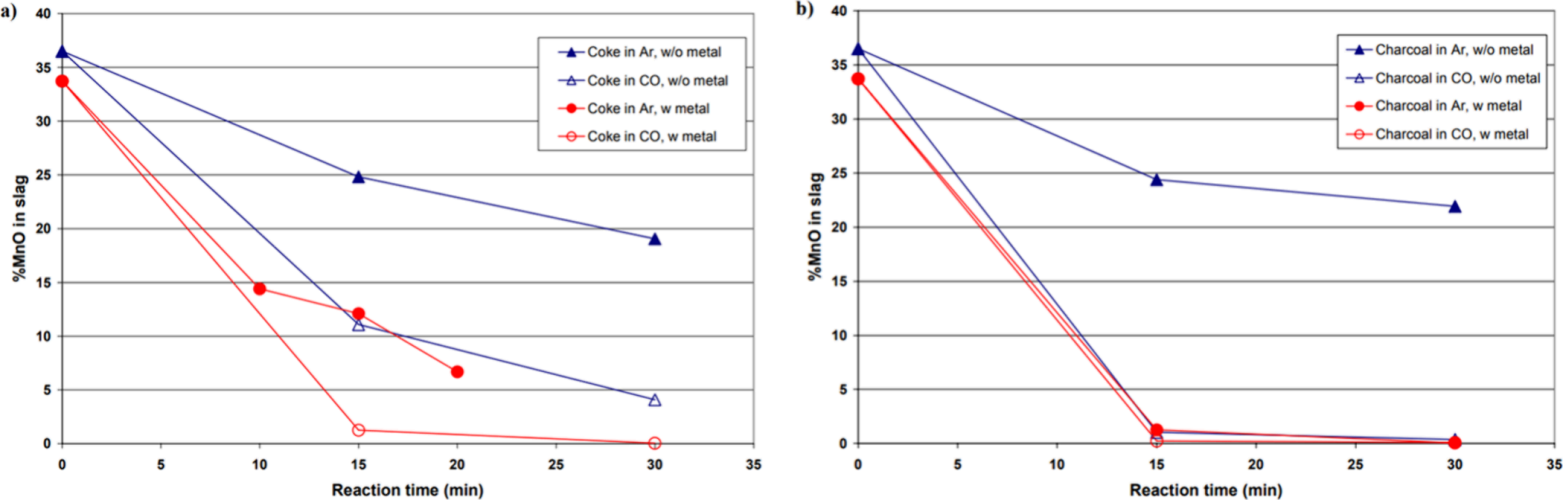
Concentration of MnO in reacted slag as a function of time for experiments (a) using coke as substrate and (b) using charcoal as substrate. Reprinted with permission from ref (86). Copyright 2007 International Committee on Ferro-Alloys (ICFA).

In addition to the reductant itself, evidence also shows that the presence of metal on the reaction surface also affects the reactivity of carbon and slag.^85,86^ Safarian et al.^85^ demonstrated when FeO is present in the Mn-slag, a metal phase could be maintained at the slag/carbon interface, and it has a positive impact on the rate of MnO reduction through metallothermic reduction by Fe and the dissolved carbon. In the study of Tranell et al.,^82^ coke and charcoal were used as carbon substrate to conduct the sessile drop experiment. The slag reactivity was analyzed by the reduction of MnO. The result is shown in Figure 15. The figures indicate that there is a notable difference in the reduction rate between slags initially containing metal and those that do not. The reduction rate is observed to be faster when metal is present at the beginning of the reduction process. It was concluded that this can be attributed to several factors: (1) The carbon-saturated micron-sized metal particles present in the slag provide a large surface area for the reaction to occur, facilitating a faster reduction rate. (2) The catalytic effect of the metal on the reduction of MnO can also contribute to this phenomenon.

### 2.4 SiO Reactivity

In the smelting process of silicon and ferrosilicon, silicon monoxide (SiO) is one of the main gaseous constituents, which is generated in the low part of the furnace where the temperature is high.^87^ The conversion of SiO_2_ to SiO requires high amounts of energy. If SiO escapes from the furnace, then it leads to decreased silicon yield and a substantial increase in power consumption.^88^ Most of the SiO gas will ascend through the furnace and react with carbon reductant to produce solid siliconcarbide. The reaction is shown as eq 5. The silicon carbide is usually regarded as the obstacle in slag processes, as the proceeding of reduction as it forms a blockage on the surface of the carbon particles.^89^ In the silicon and ferrosilicon process, the SiC production is however beneficial as it captures the SiO gas in the furnace. Considering the implications, the reactivity of SiO with biocarbon in the process of silicon and ferrosilicon production is important. This consideration aims to minimize energy consumption and enhance silicon recovery. As a summary, the research results suggest that charcoal exhibits higher reactivity compared to fossil fuels.^90,91^
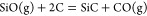
5

**Figure 16:**
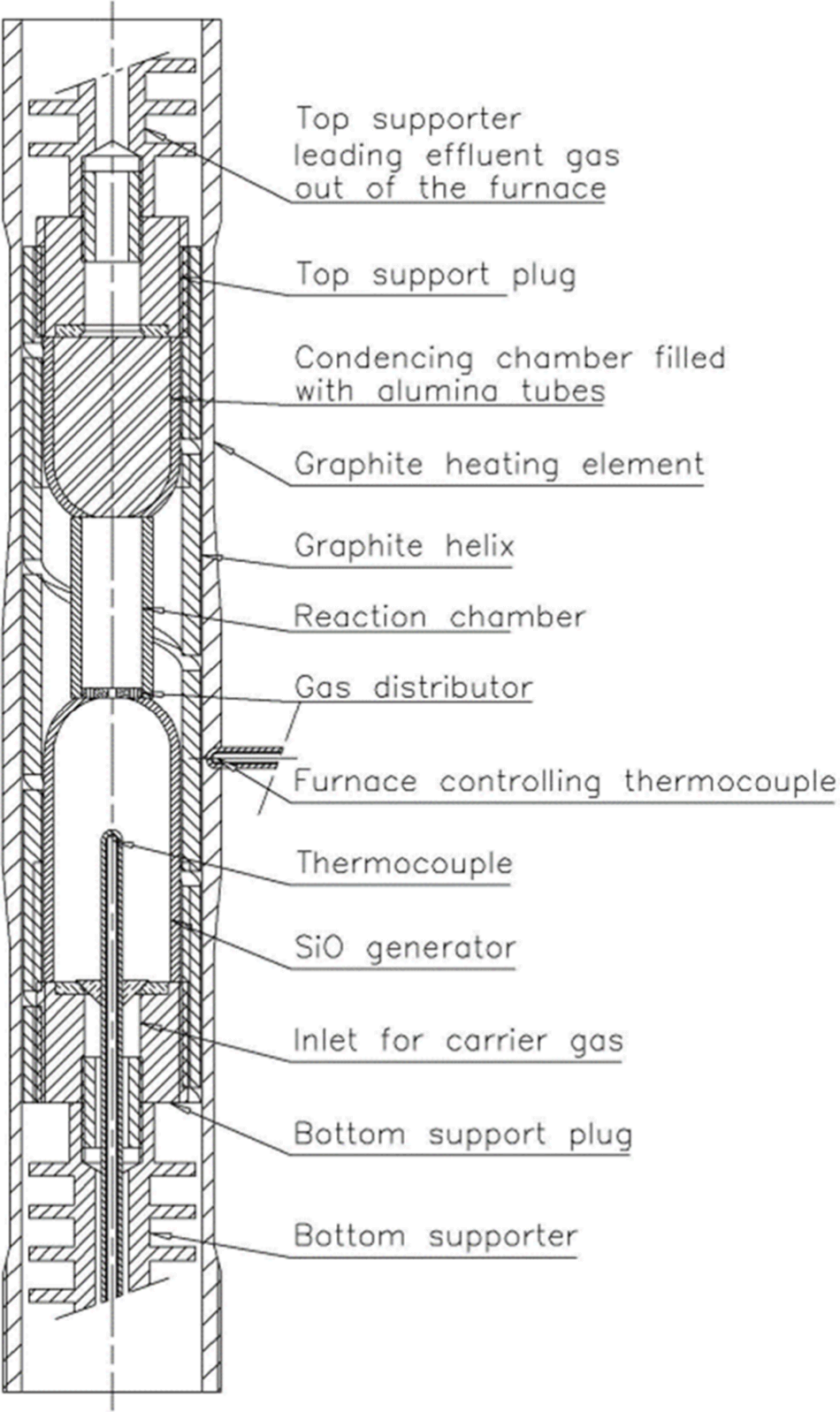
SINTEF-SiO reactor for measuring SiO-reactivity. Reprinted with permission from ref (93). Copyright 2007 International Committee on Ferro-Alloys (ICFA).

The research on reaction behavior of silicon monoxide gas and carbonaceous reducing agents is usually conducted by a technique method developed by Tuset and Raaness in SINTEF.^92^ In this method, Ar gas carries the mixture of SiO and CO gases, which are produced by a reaction between SiO_2_ and SiC. Then, the mixed gas goes through a carbonaceous, which is precalcined and sieved. The composed gas before the reaction is already known, and the generated CO gas by the reaction can be examined by the gas analyzer. Lindstad et al.^93^ introduced a correction formula to account for changes in CO content and updated certain parameters as part of their enhancements. The SiO reactor is shown in Figure 16.

**Figure 17:**
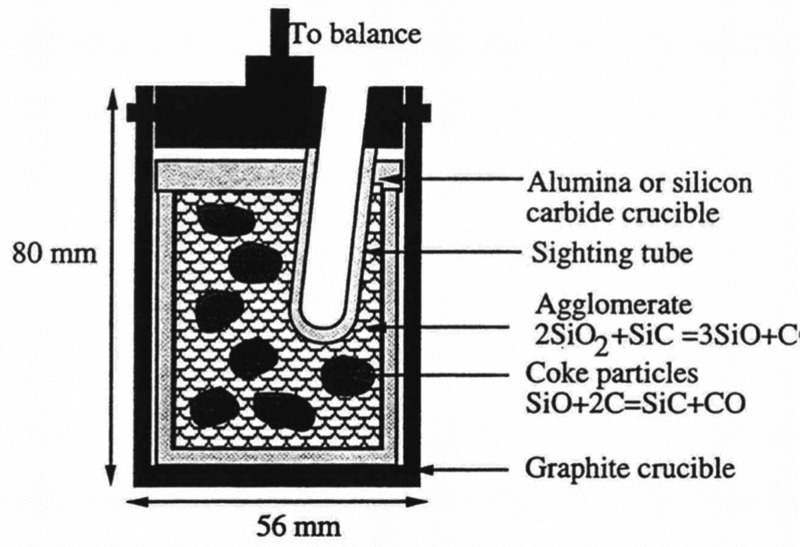
Principle for measuring reactivity of SiO in Elkems reactivity test. Reprinted with permission from ref (94). Copyright 1995 International Committee on Ferro-Alloys (ICFA).

Researchers from Elkem have also developed a method to measure the reactivity of SiO.^94^Figure 17 illustrates the principle of the testing method. In this method, the agglomerate acts as a SiO source, which is made from a mixture of fine ground quartz and silicon carbide. The experimental results are presented in terms of weight variations, calculated as the change in weight per minute (Δ*m*/Δ*t*) and expressed in milligrams per minute (mg/min).

**Figure 18:**
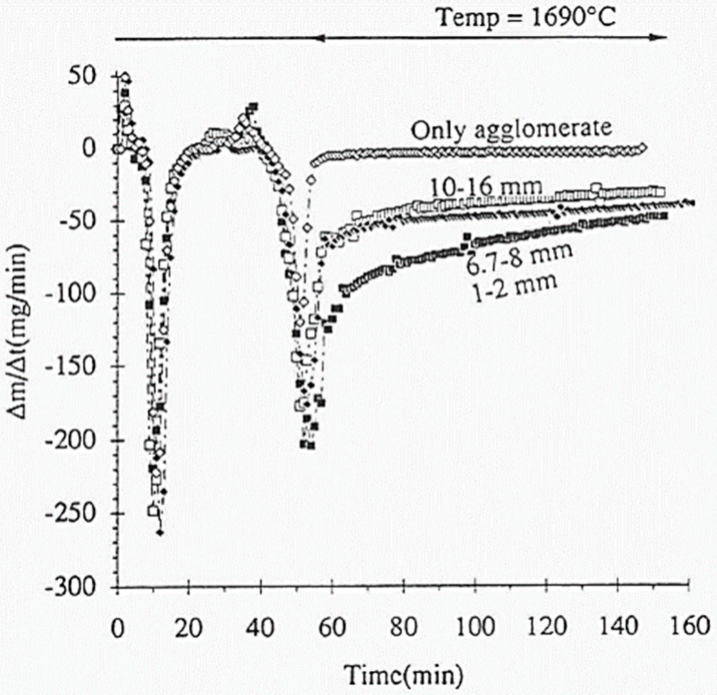
Weight variation (Δ*m*/Δ*t*) as a function of time for grain sizes of coke. Reprinted with permission from ref (94). Copyright 1995 International Committee on Ferro-Alloys (ICFA).

Paull et al.^95^ studied the interaction of SiO and green reductants and metallurgical coke. They used charcoal, petroleum coke, Iscor coke, and Lurgi char as the objects of the study. It was concluded that the charcoal has the highest SiO reactivity, followed by Lurgi char and metallurgical coke. The petroleum coke had the lowest reactivity toward SiO gas. Ramos et al. also investigated the SiO reactivity of different charcoals and coke.^96^ It was concluded that all charcoals have significantly higher reactivity than coke. In addition, the SiO reactivity shows a development trend with the decrease of wood apparent density as well as fiber wall area of materials. Li^97^ also emphasized that porosity is a significant property that impacts reactivity. The findings revealed that charcoal demonstrated higher reactivity and porosity in comparison to coke, black carbon, and coal. Specifically, the reactivity was expressed as conversion rate of SiO, which were determined to be 87.7% for charcoal, 85.9% for coke, 56.5% for black carbon, and 46.5% for coal. Apart from these factors, the particle size also has impact on SiO reactivity of reductant. In the study on the effect of particle size,^94^ the results show that reaction rate was highest for the fraction 1–2 mm, followed by the fraction of 6.7–8.0 mm and the fraction of 10–16 mm. Figure 18 shows the results of experiments on different cokes and different particle sizes.

**Figure 19:**
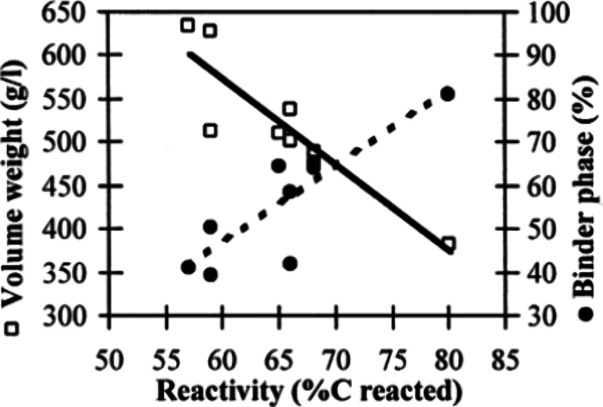
Relation of reactivity to binder phase and volume weight. Reprinted with permission from ref (98). Copyright 2000 Elsevier.

The structure of reductants is closely related to their SiO reactivity. Buo et al.^98^ utilized the petrographic composition to reflect reactivity of reduction material. The results indicated that the reaction rate could be influenced by gas diffusion and the surface area of carbon exposed to the gas. Moreover, materials with higher porosity and permeability exhibited a larger surface area, facilitating easier diffusion. Figure 19 shows the relation of reactivity and volume weight. The straight lines represent regression lines, and it can be observed that reactivity increases as the volume weight decreases and binder phase increases. As for the investigation of the relationship between reactivity and degree of anisotropy, it was found that the most reactive coke has the lowest degree of anisotropy, which is in line with the results of Raaness, Gray, and Patalsky,^99,100^ who also demonstrated the reactivity decreases with improved degree of anisotropy.

For the purpose of further exploring the reaction mechanisms of carbon materials and SiO gas, a kinetics model has been established. Myrhaug et al.^101^ agreed that the shrinking core model is the most suitable for describing the behavior of carbonaceous particles in an atmosphere containing SiO gas. This conclusion is based on thermogravimetric experiments and the SINTEF SiO-reactivity method. The optimal range of particle diameters for this model spans approximately 5 to 25 mm. The relationship between degree of conversion and time in the model of shrinking the unreacted core was described by some Sohn et al. and Szekely et al.,^102,103^ which was shown as eq 6:
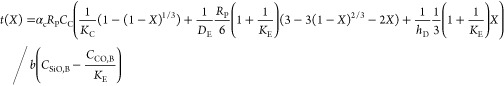
6

In this equation, several variables and constants are defined as follows:

*k*_C_: the chemical reaction rate constant in m/s;

*D*_E_: the effective gas diffusivity in the product layer, the unit is m^2^/s;

*h*_D_: the mass transfer coefficient from the gas to the solid surface in m^2^/s;

*K*_E_: the equilibrium constant;

*R*_P_: the radius of the sphere;

*b*: the number of C(s) moles;

*C*_SiO,B_: the concentrations of SiO(g);

*C*_CO,B_: the concentrations of CO(g) ;

α_C_: volume fraction of solid C(s)

*C*_C_: molar density of solid C(s).

*X* is the degree of conversion of C(s) to SiC(s) in the particle, which can be calculated as eq 7:
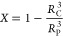
7

Here, *R*_C_ represents the radius of the solid C(s).

### 2.5 Mechanical Strength

Prior to the smelting process, it is important to consider the mechanical strength of the charge. Low strength of the raw materials will create fines during the long-term storage and long-distance transport.^104^ The mechanical strength can be correlated to the pulverization rate or the decrepitation rate, as both terms are used in literature. If the strength is too low, then the pulverization degree of the material is high. This excessive pulverization negatively impacts the permeability of the furnace, hindering its efficient operation and leading to increased energy consumption.^105^ Therefore, the mechanical strength of the carbonaceous reductant is a significant factor to consider.

According to different requirements and materials, the strength can be assessed by abrasion resistance, friability, tensile strength, or compressive strength.^106^ Each of these strength characteristics requires specific testing methods to accurately evaluate the material’s performance.

The compressive strength is employed by researchers and industrial participants widely to reflect the materials’ cold resistance. To calculate the compressive strength of the sample, the ratio of the applied pressure to the sectional area of the sample is determined.^107^ Kaffash et al.^105^ measured the compressive strength of charcoals, which were treated in different ways. The results are shown in Table 8. The compressive strength of untreated charcoal particles ranged from 1.54–5 MPa, but the compressive strength of three kinds of densified charcoal is 5 to 10 times higher than that of the original charcoals, which ranged from 10 to 27 MPa, which is close to the compressive strength of the metallurgical cokes (20–30 MPa). It suggested that the metallurgical coke can be replaced by densified charcoal from the view of compressive strength.

**Table 8:** Compressive Strength of Charcoals and Cokes^108^

sample	compressive strength (MPa)		
	raw	heat treated in Ar	densified
charcoal A	2.8–4.60	3–9.3	16–27
charcoal B	1.54–3	4.54–7.29	10–14.7
charcoal C	3–5	3.5–5	12.2–13.4
metallurgical coke	20–30		

**Figure 20:**
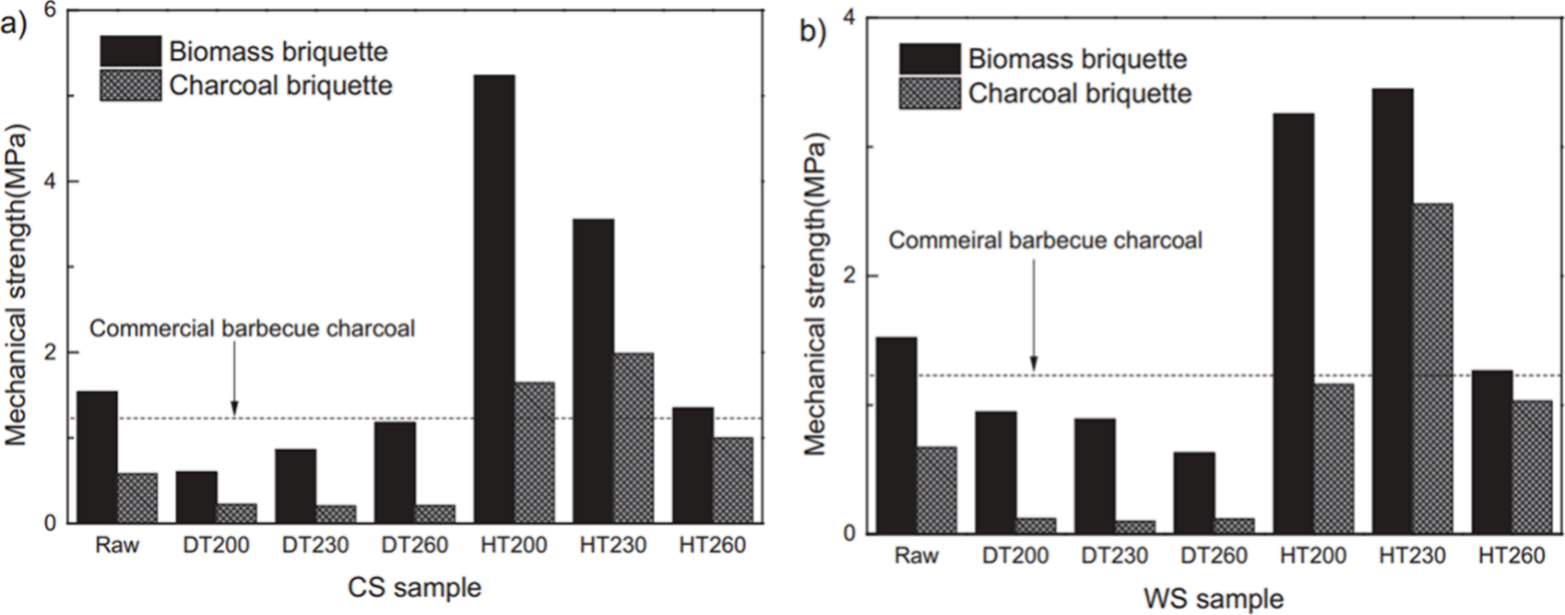
Compressive strengths of the biomass briquette and charcoal briquette. (a) Cotton stalk sample. (b) Pine wood sawdust sample. Reprinted with permission from ref (109). Copyright 2018 Elsevier.

Wu et al.^109^ measured the compressive strengths of biomass briquettes densified at 200–260 °C, and charcoal briquettes which were carbonized from the former biomass briquettes at the temperature of 400 °C. It can be seen in Figure 20 that the compressive strength of the biomass is at a much higher level. Meanwhile, the biomass briquette proceeding hydrothermal treatment (HT) has a higher compressive strength than the raw and dry-torrefied biomass (DT) briquettes. It was believed that high compressive strength of densified material is attributed to strong bonding force that bound the particles together. The presence of extremely high compressive strength in HT (hydrothermally treated) biomass briquettes indicates the existence of strong bonding forces between the particles.

**Figure 21:**
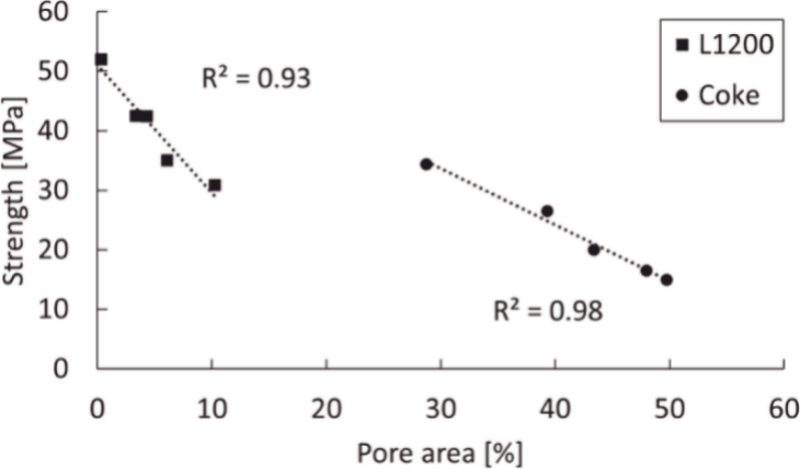
Relationship between compressive strength and pore area. Reprinted with permission from ref (110). Copyright 2023 Elsevier.

Some researchers also conducted investigations of the relationship between compressive strength and other physical properties. Koskela et al.^110^ observed that the increase in the pore size is correlated with the strength of both coke and pyrolyzed lignin biocarbons after gasification (L1200: pyrolysis temperature: 1200 °C), which can be seen in Figure 21. They also explained that the strength and pore area of the samples primarily depend on the surface characteristics of the carbon matrix. In this case, the L1200 briquette, which has a dense carbon matrix, exhibits higher strength properties compared to coke after the gasification process. The investigation results from Kumar et al.^111^ proved that the reduction in pore size may increase the rigidity of charcoal as well as its compressive strength. Dufourny et al.^112^ adopted the stability index *S* (the percentage of charcoal retained in a given sieve relative to the initial mass; %), to assess the strength of different charcoals. In the study conducted, it was found that spruce charcoal (*S*: 85.4–94.0%) exhibited higher compressive strength compared to eucalyptus charcoal (*S*: 64.9–77.7%), regardless of the pyrolysis conditions (both charcoals pyrolyzed at temperature of 500 and 800 °C). Additionally, the study observed a linear relationship between the *S* index and the true density of spruce charcoal. This finding suggests that there is a strong correlation between the compressive strength of charcoal and the ordering and density of its carbon structure.

Except for compressive strength, the abrasion thermal stability is also often used for analyzing the mechanical quality of bioreductants, a method for measuring the abrasion strength involves subjecting the reacted carbonaceous material to a tumbling process.

The char strength after reaction (CSR) is often mentioned together with the reactivity index (CRI) (CRI is illustrated in section 2.1, CO_2_ reactivity) for assessing the thermal abrasion ability of char. After completion of the CO_2_ gasification, the char sample is cooled under N_2_ atmosphere. The mass of gasified samples is weighed and subjected to a drum at a total of 600 revolutions. Then, the CSR equation^42,43^ can be obtained as follow:
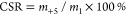
8

The *m*_1_ is the char mass after reaction and *m*_+5_ is the char mass of the +5 mm particles after the drum test.

Since CSR depends on CRI, NTNU, and SINTFE proposed an improved procedure,^30^ where an atmosphere composed of 50% CO and 50% CO_2_ is used. The abrasion strength is assessed independently of CO_2_ reactivity, given that roughly 20% of the fixed carbon is consistently reacted in all samples. The sample is placed in a Hannover drum and tumbled for 30 min at a speed of 40 rpm. Then, the cohesion index (C.I.) and the thermal stability index (T.I.3) are analyzed after tumbling. The specific definitions and instructions of two indices were illustrated as C.I. means cohesion index, and it is characterized by the fraction larger than 4.75 mm before tumbling but after the CO_2_ reactivity test; T.I.3 represents the fraction larger than 3.33 mm after tumbling for expressing the index of thermal stability. In addition, in their research,^34^ the C.I of the industrial charcoals from Brazil is from 74% to 84%. The cohesion strength of preserved wood charcoal is around 93–95%. Which is similar to coke (93–97%). For the T.I.3 index, there is a smaller difference between charcoal (78–82%) and coke (82–89%).

**Figure 22:**
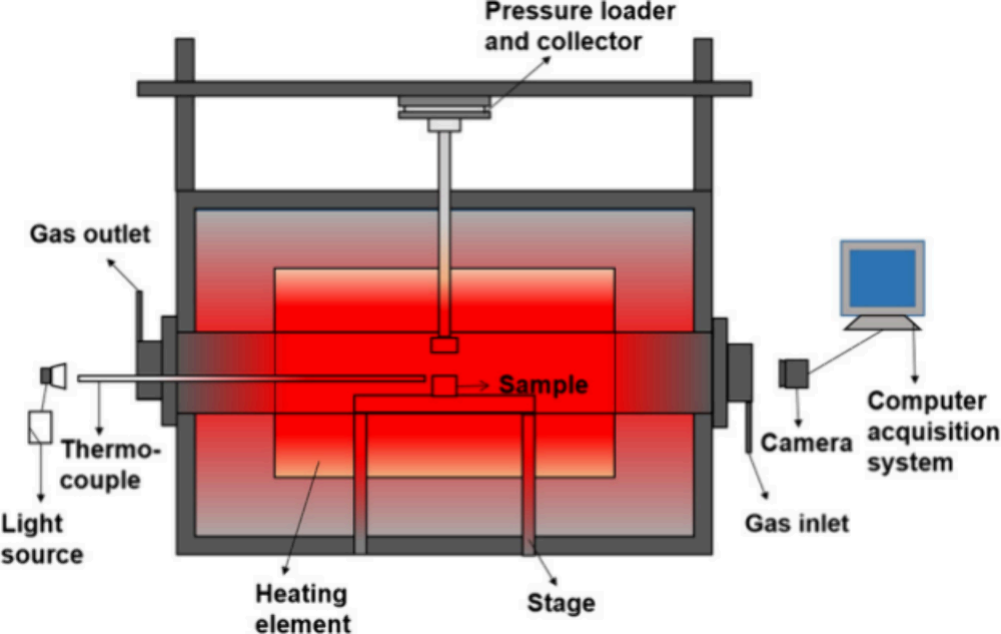
System of measuring equipment. Reprinted with permission from ref (113). Copyright 2020 Springer Nature.

The thermal crushing strength is also an assessment for determining the utilization ability of charcoals. Wuhan University of Science and Technology and Anshan Xingyuanda Co., Ltd. developed a particular setup to examine it.^113−115^ The system of equipment was exhibited in Figure 22. The samples underwent the test during the gasification for a specific duration. Then, a zirconia pressure bar dropped down at the rate of 0.5 mm/min. The pressure value at breakpoint was recorded as the result. It was found that the thermal crushing strengths of gas-coal coke (CK), ferro-coke (FCK), and modified ferro-coke (PFCK) were 1588.4 N, 410.8 N, and 510.0 N, respectively, before the gasification reaction. In addition, the crushing strength of cokes declines with the gasification time. For this reason, it was explained that as the gasification reaction proceeded, the flat surface of coke gradually became rough and thick, which is attributed to the generation of large pores and the presence of residual ash caused by the gasification process.

**Figure 23:**
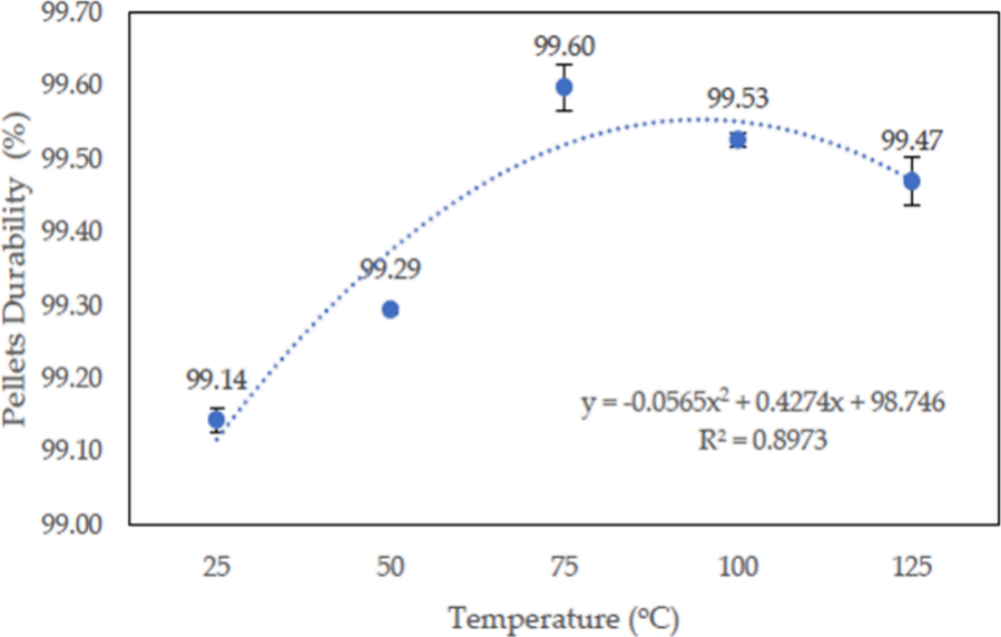
Impact of temperature on the biocarbon pellets’ mechanical durability. Reprinted with permission from ref (116). Copyright 2023 MDPI.

Ismail et al.^116^ conducted a durability test on Khaya senegalensis pellets. The pellets underwent shaking in a sieve shaker for 10 min at 50 rpm. After this procedure, the intact pellets were weighed to ascertain their final mass. The outcomes are illustrated in Figure 23. The obtained values satisfy the Pellet Fuels Institute (PFI) standard^117^ which illustrates that the durability of premium quality pellets should be a minimum of 96.5%. It is obvious from Figure 23 that with increasing temperature from 25 °C, the durability experiences a gradual rise. However, a declining trend is observed after the temperature further elevates to 100 and 125 °C. They explained that under elevated temperatures, biomass constituents like lignin, hemicellulose, cellulose, and protein become activated between particles, leading to increased particle adhesion.^117^ However, higher temperatures cause the pellets to attain greater density and heightened durability due to the raw materials becoming liquid and then hardening upon cooling.^118^ The decrease in durability can be attributed to the belief that excessive temperature may result in the melting of lignin, thereby reducing particle plasticity. Furthermore, excessive denaturation of proteins could lead to decreased particle durability.^119^

## 3 Application of Biocarbon in Ore Sintering Process

The manganese ore fines cannot be added into the submerged arc furnace (SAF) due to causing poor gas permeability.^120^ As a result, the manganese ore fines must undergo an agglomeration process to form agglomerates with specific sizes. It is widely acknowledged that sintering can enhance the practical properties of the charge, leading to energy and cost savings.^121^

The amount of carbon in the sintering mix is a crucial parameter when producing sinters, as it largely determines the properties of sinters. If it is too low, then the amount of liquid phase generated inside the sinters will be scarce, resulting in serious decrepitation when it is fed into the furnace and a sharp decrease in the permeability of the charge. If the carbon content is too high, then there will be too much liquid phase, which is not conducive to the subsequent reduction process of the sintered products in the SAF.^122−124^ Zhang et al.^125^ studied the effect of carbon addition on the properties of manganese sinters on laboratory scale and discovered that a carbon content of approximately 9.9% resulted in the highest tumbler strength of the sinter, reaching 54.5%. Additionally, when the carbon content is 9.6%, the yield of sinter was the highest (77.6%). Dmitriev et al.^126^ also showed that sintered product with Dzhezdinsk and Polynochnyi ore has the highest strength when the coke content is 9 wt %. Han et al.^127^ also discussed the effect of carbon content and basicity on the performance of sinters, and it was concluded that the optimal binary basicity () should be 0.7. The strength of manganese sinter increases with the addition of coke and when the carbon content is 10%, the sinter tumbler strength is reaching the maximum (75.33%).

**Figure 24:**
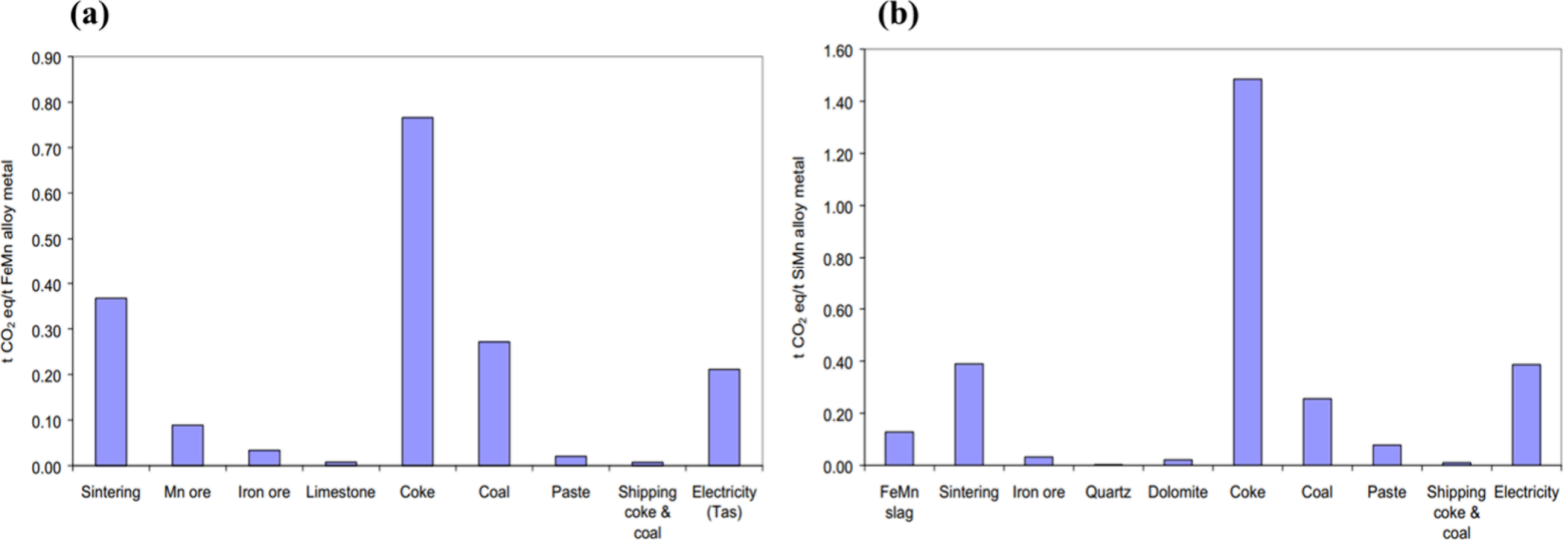
Contribution of greenhouse gas emissions from materials and energy components of FeMn and SiMn production. (a) FeMn production. (b) SiMn production. Reprinted with permission from ref (130). Copyright 2013 Elsevier.

The conventional carbon material is coke breeze in the process of sintering. Nevertheless, it is important to acknowledge the emissions associated with the sintering process due to the considerable use of coke. Some studies have highlighted the significant generation of undesirable gases such as CO_2_, SO_*x*_, and NO_*x*_ during the combustion of fossil carbon in ore sintering. The iron-ore sintering process contributes up to 10% of the overall mass of carbon dioxide released from an integrated iron and steel works.^128^ In China, the NO_*x*_ produced in iron ore sintering amounts to the major NO_*x*_ emissions in the integrated iron and steel works, accounting for around 6% of total NO_*x*_ release.^129^ The circumstance in Australia, Nawshad, and Terry addressed the significant contribution of the sintering process to CO_2_ emissions in the production of ferromanganese (FeMn) and ferrosilicon (SiMn) alloys. Figure 24 presents the breakdown of CO_2_ emissions throughout the production processes of these two alloys. It is evident that the sintering process alone contributes approximately 0.38 t CO_2_ eq/t FeMn alloy and 0.4 t CO_2_ eq/t SiMn alloy.^130^ In the study of Luke et al.,^131^ a Life Cycle Assessment (LCA) modeling approach was employed to investigate various potential environmental impacts and indicators associated with the manganese alloy production chain. The study revealed that stage of mining and sintering plays significant roles in multiple environmental impacts and indicators. Furthermore, these processes are responsible for 99% of the waste generated (by mass) from primary manganese processes.

Consequently, it is urgent to replace the fossil fuels with biocarbon in the sintering process. Marian’s team studied the effect of the addition of biochars, which is a fuel mixture composed of charcoals, on the quality of sinters.^132^ They found the application of biochars can lower the FeO content in sinters, which makes the reducibility of sinters much better as it will consume less coke in the reduction process. Considering productivity, fuel consumption, and properties of the sinter, it was concluded that the best ratio of biochar in fuel should be at the range from 10 to 30 wt %. Kieush et al. studied the influence of different biomaterial types (sunflower husk, walnut shell, and charcoal) on the process of iron ore sintering.^133^ They addressed that it is feasible to replace 25% of coke breeze by charcoal or walnut shell. It was also suggested that the crucial characteristic of these biomaterials used as fuel for sintering is their bulk density, which determines the thermal parameters of the sintering process, as well as the resulting sinter structure to a large extent. Other studies^134−136^ also had the similar conclusion that the optimal amount of biofuel additive mixed with coke breeze is 20–30%. These findings are instructive for the application of biochar in manganese ore sintering. However, it should be noted that manganese ore sintering requires more carbon content compared with iron ore sintering, as higher combustion loss and heat consumption associated with the characteristic of manganese occur.^137,122^

MnO_2_ and Mn_2_O_3_ are the main manganese oxides in manganese ore. These oxides are transformed into Mn_3_O_4_ during the sintering process. Subsequently, in the mid part of the submerged arc furnace (SAF), all manganese oxides are further reduced to MnO. Finally, carbon directly reduces MnO to produce metallic manganese.^138−141^ Braga et al. carried out a series of experiments by adding charcoal as reductant to analyzing the self-reduction of the Mn pellets,^142^ and they expressed that it is possible to obtain the alloy with good recovery yield of Mn at temperatures around 1300–1400 °C, without disintegration of the charcoal bearing pellet. It was also mentioned that the productivity would be improved 10% and the power would be decreased by 380 kWh^–1^ of alloy by replacing lump ore with this kind of self-reducing agglomerates for smelting commercial Fe–Si–Mn alloy. In a specific study conducted by Suharto et al.,^143^ manganese pellets were prepared by incorporating palm shell charcoal at a weight equivalent to 25% of the manganese ore. The primary objective of this research was to examine the influence of temperature on the reduction process of manganese oxides. According to the results, the highest content of manganese (41.28 wt %) was obtained by roasting 120 min at a temperature of 1100 °C.

Regarding the process of the manganese ore sintering, Kieush et al. used initial and prepyrolyzed coniferous wood to study the optimal amount and pyrolyzed temperature of biobased fuel (coniferous wood).^143^ The findings indicate that in order to achieve a similar specific capacity and yield as when using only coke breeze for sinter production, the amount of biofuel used in manganese ore sintering should be kept below 25 wt % of the solid fuel. Additionally, it was observed that the biofuel needs to undergo prepyrolysis at a temperature of 1273 K. Zhang et al.^144^ replaced coke with biomass fuel in the lab-scale sintering process of manganese ore. In their results, the tumbling index of using the mixed fuel with 40 wt % biomass charcoal declined by 5% compared with that of using coke, whose tumbling index was 61%. However, when 20% of biomass was mixed with fuel, the highest sintering yield can be reached (84%). For the productivity, the highest productivity of 1.6 t/(m^2^h) can be achieved when 30% of biomass was used.

As described in the previous discussion, biocarbon generally exhibits higher volatile matter content, increased porosity, and enhanced gas reactivity compared to conventional fuels. These unique characteristics will cause more combustion and gas reaction in the sintering process,^145^ which may influence the sintering process as well as the quality of products. Indeed, it also has advantages. The findings presented by Zandi et al.^146^ indicate that the composition of biomass, specifically the proportions of cellulose, hemicellulose, and lignin, exerts an influence on the combustion behavior within the sinter bed. Therefore, a notable trend emerges where the temperature rise tends to occur earlier by using some amount of biomass compared to coke breeze, and the thermal profiles tend to exhibit greater width compared to those observed with coke breeze alone. Similarly, in line with the observations made by Machado et al.,^147^ the sintering process is slightly accelerated when the mixture includes biomass or charcoal, in contrast to the sintering tests involving coke breeze alone. Meanwhile, numerous investigations have demonstrated the feasibility and reliability of replacing certain amounts of coke with biobased fuels in various industrial processes, including the production of Mn-alloys. This presents a potential pathway for the application of biocarbon in the agglomeration process of manganese ore.

## 4 Conclusions and Outlook

Biocarbon, as an eco-friendly and naturally derived material, has a substantial developmental potential. It can serve as a substitute for metallurgical coke in metal production, effectively contributing to the reduction of carbon emissions. Indeed, the metallurgical properties of biochar are closely linked to its chemical composition and physical characteristics such as porosity, surface area, and internal structure. However, its limited mechanical stability poses challenges for long-distance transport and charging into enclosed furnaces. Its elevated volatile matter content, higher CO_2_ reactivity entail risks for the submerged arc furnace (SAF) smelting process. These concerns, however, can be adeptly addressed through techniques such as densification, pyrolysis, carbonization, and agglomeration, improving these shortcomings. In addition, numerous studies have shown that substituting specific quantities of coke with biobased fuels into the agglomeration process of manganese ore can lower the sintering temperature and enhance the quality of products, which proves the dependability and benefits of using biocarbon in the metallurgy field.
